# The HelQ human DNA repair helicase utilizes a PWI-like domain for DNA loading through interaction with RPA, triggering DNA unwinding by the HelQ helicase core

**DOI:** 10.1093/narcan/zcaa043

**Published:** 2021-01-12

**Authors:** Tabitha Jenkins, Sarah J Northall, Denis Ptchelkine, Rebecca Lever, Andrew Cubbon, Hannah Betts, Vincenzo Taresco, Christopher D O Cooper, Peter J McHugh, Panos Soultanas, Edward L Bolt

**Affiliations:** School of Life Sciences, The University of Nottingham, NG7 2UH, Nottingham, UK; School of Life Sciences, The University of Nottingham, NG7 2UH, Nottingham, UK; Department of Molecular and Cell Biology, University of Leicester, LE1 7RH, Leicester, UK; Research Complex at Harwell (RCaH), OX11 0FA, Didcot, UK; MRC Weatherall Institute of Molecular Medicine (WIMM), University of Oxford, OX3 9DS, Oxford, UK; School of Life Sciences, The University of Nottingham, NG7 2UH, Nottingham, UK; School of Life Sciences, The University of Nottingham, NG7 2UH, Nottingham, UK; School of Chemistry, The University of Nottingham, NG7 2RD, Nottingham, UK; School of Pharmacy, The University of Nottingham, NG7 2RD, Nottingham, UK; Department of Biological and Geographical Sciences, School of Applied Sciences, The University of Huddersfield, HD1 3DH, Huddersfield, UK; MRC Weatherall Institute of Molecular Medicine (WIMM), University of Oxford, OX3 9DS, Oxford, UK; School of Chemistry, The University of Nottingham, NG7 2RD, Nottingham, UK; School of Life Sciences, The University of Nottingham, NG7 2UH, Nottingham, UK

## Abstract

Genome instability is a characteristic enabling factor for carcinogenesis. HelQ helicase is a component of human DNA maintenance systems that prevent or reverse genome instability arising during DNA replication. Here, we provide details of the molecular mechanisms that underpin HelQ function—its recruitment onto ssDNA through interaction with replication protein A (RPA), and subsequent translocation of HelQ along ssDNA. We describe for the first time a functional role for the non-catalytic N-terminal region of HelQ, by identifying and characterizing its PWI-like domain. We present evidence that this domain of HelQ mediates interaction with RPA that orchestrates loading of the helicase domains onto ssDNA. Once HelQ is loaded onto the ssDNA, ATP-Mg^2+^ binding in the catalytic site activates the helicase core and triggers translocation along ssDNA as a dimer. Furthermore, we identify HelQ-ssDNA interactions that are critical for the translocation mechanism. Our data are novel and detailed insights into the mechanisms of HelQ function relevant for understanding how human cells avoid genome instability provoking cancers, and also how cells can gain resistance to treatments that rely on DNA crosslinking agents.

## INTRODUCTION

The Ski2-like family of RNA and DNA helicases includes the HelQ DNA repair helicase (1). Deletion of the *helq* gene (Δ*helq*) causes genome instability, an enabling factor triggering cancers (2). This is manifest through multiple phenotypes caused by loss of HelQ function in human, mouse, *Drosophila* and *Caenorhabditis elegans*: Sensitivity to the DNA inter-strand crosslinkers (ICLs) mitomycin C (3–5), nitrogen mustard (6,7) and cisplatin (8), although sensitivity to mitomycin C was absent from chicken DT4 Δ*helq* cells (9); sensitivity to camptothecin (10) that blocks DNA replication by targeting DNA topoisomerase I; increased replication fork stalling in the absence of ICLs (5); accumulation of radial chromosomes (3); defects in oocyte (11) and sperm development (4,5); and defective meiotic DNA double-strand break repair (12). In human cells, phenotypes caused by Δ*helq* were made more severe when additional deletions were made in Fanconi anaemia genes (5) or in HROB-MCM8 (8), indicating that HelQ functions in human DNA repair that is distinct from those systems. However, similar genetic combinations in worms indicated that *helq* is epistatic with *fcd-*2, encoding FancD2 (6) and additionally that *helq* is epistatic with *jmjd*-5 that encodes a histone-modifying enzyme (13).

These genetic problems associated with Δ*helq* highlight the role of HelQ helicase as one of the ‘caretakers of the genome’, defects in which pre-dispose cells to becoming cancerous (14,15). GWAS (Genome-Wide Association Studies) have identified *helq* polymorphisms and susceptibility to cancers of the upper aerodigestive and respiratory tract and breast (16–18). In U2OS cells cancer phenotypes are triggered by Δ*helq* (19), and copy number variations in *helq* are associated with ovarian cancers (3,20). In addition, over-expression of HelQ promotes resistance to treatments for ovarian cancers that are based on use of DNA crosslinkers (21,22), implicating HelQ levels in cells as potential factor to be overcome in hard-to-treat cancers. Overall, genetic analysis of *helq* and cancer association studies implicate HelQ helicase in recovery of DNA replication that is stressed or broken by endogenous or environmental stresses, arising in ways that are reviewed recently in references (23,24).

Human HelQ protein was isolated and characterized as a ssDNA-dependent adenosine triphosphatase (ATPase) that translocates DNA with 3′ to 5′ polarity (25). It unwinds DNA duplexes especially well within forked DNA substrates and was observed to co-localize with replication forks that had been blocked by camptothecin treatment, where it persisted for >8 h (26). DNA replication that is inactivated by DNA breaks or lesions can be restarted by homologous recombination with essential roles for DNA helicases, recently reviewed (27–31), or by ‘skipping’ the block and re-priming replication downstream of the lesion (32–34). Generation of ssDNA is prominent during these events, when new DNA synthesis is halted but DNA unwinding continues and when ssDNA is utilized for repair by homologous recombination and for resuming replication past the lesion or break. The replication protein A (RPA) in eukaryotic cells is an essential ‘first responder’ to ssDNA arising, see reviews (35,36). This protects DNA and through dynamic interactions with RPA, and multiple other proteins RPA controls for subsequent repair processes (37–39). HelQ and RPA interact in human cells (3,4) and RPA stimulates HelQ helicase assays *in vitro* by a mechanism unknown, but not through preventing re-annealing of unwound DNA strands (26). Here, we present new data identifying that a conserved fold in human HelQ triggers displacement of RPA from ssDNA, suggesting how HelQ loads onto ssDNA at stressed replication forks, and revealing why RPA stimulates HelQ helicase activity. Furthermore, we provide new molecular details showing that when loaded onto ssDNA HelQ is activated by ATP-Mg^2+^ for translocation as dimer, requiring interaction with DNA bases.

## MATERIALS AND METHODS

### DNA substrates

DNA substrates and details of their chemical modifications are illustrated in Supplementary Figures S1A and B. Unmodified DNA oligonucleotides were purchased from SIGMA with Cy5 label on 5′ ends. Chemically modified oligonucleotides were purchased from Eurofins (phosphonothioate DNA), EuroGenTech (Abasic DNA, formed as a stable tetrahydrofuran) or IDT (methyl phosphonate DNA). Unless stated, enzymes for DNA manipulations were from New England Biolabs (NEB). Forked DNA was made by annealing to room temperature overnight a 1.2:1 ratio of unlabelled to Cy5-labelled oligonucleotides, after heating at 95°C for 10 min. DNA substrates were separated from unannealed oligonucleotides by gel electrophoresis through 10% w/v acrylamide Tris.HCl, borate and ethylenediaminetetraacetic acid (EDTA) (TBE) gels, followed by excision of the desired substrate as a gel slice gel and soaking the slice overnight into Tris.HCl pH 7.5 + 150 mM NaCl to recover DNA. G4 Quadruplex was formed from a 50mer oligonucleotide following the method in (40), and was 3′ end-labelled using Aminoallyl-UTP-Cy5 (Jena Bioscience) incubated with TdT.

### Proteins

HelQ and the N-terminal HelQ fragment (N-HelQ) were over-expressed using the Bac-to-Bac baculovirus expression system from Gibco BRL in SF9 insect cells, each with N-terminal (His)_6_, SUMO and StrepII-Flag tags (25). SF9 cells were cultured in LONZA Insect Xpress protein-free culture media with L-glutamine supplemented with pluronic acid, penicillin-streptomycin and amphotericin B. HelQ protein over-expression was optimized at 5 μl virus per 1 × 10^6^ SF9 cells for 48 h at 27°C. The N-terminal region of HelQ was produced by over-expression of HelQ for prolonged periods, typically 72 h at 27°C, resulting in natural protein degradation and the formation of a stable tagged HelQ fragment, summarized in Supplementary Figure S2. This fragment (N-HelQ) comprised HelQ amino acids 1–240 as verified by mass spectrometry. Biomasses were resuspended in lysis buffer (150 mM Tris pH8, 150 mM NaCl, 20 mM imidazole, 10% v/v glycerol) containing COMplete EDTA free protease inhibitor tablets and stored at −80°C until purification.

For purification of full-length HelQ columns and buffers were chilled to 4°C before use. Purification of the HelQ protein into fractions was followed by Coomassie staining of sodium dodecyl sulphate-polyacrylamide gelelectrophoresis (SDS-PAGE) gels. Cell biomass was thawed on ice over several hours before lysis on ice by sonication at 80% pulsed for 1 min per 5 ml biomass. Soluble proteins after sonication were collected by centrifugation at 22 000 rpm in centrifuge Avanti J-26 XP, rotor JLA 10.500. These were fractionated by precipitation using 0–50% saturation with solid ammonium sulphate added on ice with gentle agitation, at a rate of adding that was ∼0.3 g per minute. Precipitated protein was recovered by centrifugation as before, and resuspended in 50 mM NaCl, 20 mM Imidazole, 50 mM Tris pH8, 10% v/v glycerol, 1 mM TCEP to dissolve precipitated proteins. Solubilized proteins were loaded onto a 5 ml NiCl_2_ charged Ni-NTA column equilibrated with NTA-A buffer (20 mM Tris.HCl pH 8.0, 10% v/v glycerol, 1 M NaCl, 10 mM imidazole, COMplete EDTA free protease inhibitor tablet). HelQ protein eluted from this column at ∼100–200 mM imidazole was pooled and dialysed for 3 h at 4°C into 150 mM NaCl, 10% v/v glycerol, 50 mM Tris.HCl pH 8.0. Dialysed HelQ was loaded onto a 5 ml heparin column, washed in HepA buffer (50 mM Tris.HCl pH 8.0, 10% v/v glycerol, 150 mM NaCl, 1 mM TCEP, COMplete EDTA free protease inhibitor tablet) and bound HelQ eluted from heparin in a broad peak between 100–400 mM NaCl. HelQ fractions were dialysed into cold buffer HepA for 3 h and the protein was next loaded onto a 1 ml anion exchange Q-sepharose column. This column was washed in buffer HepA and HelQ eluted in a gradient of increasing NaCl concentration at ∼350–600 mM NaCl. Pooled HelQ protein was dialysed for 3 h at 4°C into 30% v/v glycerol, 5 mM dithiothreitol (DTT), 150 mM NaCl, 50 mM Tris.HCl pH 8.0 and aliquotted for storage at −20°C. Protein concentration was calculated using Bradford's reagent and a calibration curve of known concentrations of bovine serum albumin.

Purification of N-HelQ was as described above except that the heparin column step was not included because N-HelQ did not bind to it in pilot trials. C-HelQ was expressed with an N-terminal His-tag in *Escherichia coli* according to the same method as was used for N-HelQ and following the same purification procedure as described for HelQ. RPA was purified at the Research complex in Harwell following previously described methods (41), and can be seen in Supplementary Figure S3.

### ATPase assays

ATPase activity was measured using the malachite green dye assay (42). Reactions of 20 μl contained protein mixed with magnesium and ATP (5 mM each), and ssDNA that was either strand 1 from fork 2 (25 nM) or M13 (300 ng), as stated in results. Reactions were at 37°C for 10 min, concluded by addition of 80 μl of detergent-free water and 800 μl of dye reagent for 2 min at room temperature, followed by addition of a 34% aqueous solution of sodium citrate to allow colour development for a further 30 min. Concentration of liberated phosphate was measured by absorbance at 660 nm against a zero-protein blank and a calibration curve of known phosphate concentrations. Dye reagent was a 3:1 ratio of stock solutions of malachite green: ammonium molybdate. Malachite green stock was 0.045% (weight to volume) malachite green in water, and ammonium molybdate to 4.2% (w/v) in 4 M HCl.

### Protein–DNA binding and unwinding assays

DNA substrates are shown in Supplementary Figure S1. Electrophoretic mobility shift assays (EMSAs) were all in 1× buffer HB (10 mM Tris pH 7.5, 50 μg/ml bovine serum albumin (BSA) and 5% v/v glycerol) containing fresh DTT (25 mM). Protein and DNA were incubated at 37°C for 10 min in 20 μl reaction volumes. Reactions were loaded directly onto 5% w/v acrylamide TBE gels for electrophoresis at 150 volts for 2 h in Protean II tanks in 1 × TBE buffer. In EMSA reactions containing RPA and HelQ, RPA was pre-incubated with DNA at 37°C prior to adding HelQ.

Helicase unwinding assays were in the buffer HB buffer supplemented with 5 mM magnesium chloride and 5 mM ATP containing fresh DTT (25 mM). Cy5 labelled DNA substrate (25 nM) was used with addition of ‘cold-trap’ unlabelled oligonucleotide to 2.5 nM. Helicase reactions were stopped at the time indicated in results by addition of buffer STOP comprising 2.5% w/v SDS, 200 μM EDTA and 2 μg/μl of proteinase K. Reaction products were assessed after electrophoresis through 10% acrylamide TBE gels. Gels were imaged using FLA-3000 or Typhoon machine and quantified using ImageJ, GelEval and Prism software.

### Analysis of HelQ by native PAGE

Blue Native PAGE was carried out using 3–12% Bis-Tris gels, markers and loading buffers bought from Invitrogen (#BN1001BOX) as described in the manufacturer's instructions. Protein was pre-incubated in loading buffer at 37°C for 10 min and gel electrophoresis was at ambient temperature for 60–90 min at 175 volts.

### Analysis of HelQ by size exclusion chromatography and SEC-MALS

Size exclusion chromatography was using a Superdex-200 column in buffer comprising 50 mM Tris–HCl pH 8.0, 150 mM NaCl and 10% v/v glycerol, plus ATP and magnesium as stated in results. HelQ was compared to the molecular mass standards indicated in Figures 2A and 3C, bought from GE Healthcare (cat. No. 10196234). Size exclusion chromatography-multi-angle static light scattering (SEC-MALS) was also using a Superdex-200 column in the same buffers in a Wyatt Dawn 8+ 1260 Infinity II machine. Pure HelQ protein (200 μl of 2.4 μM monomer concentration) was applied to the column at a flow rate of 1 ml/min, after pre-incubated of HelQ in buffer for 10 min at 37°C. Protein mass was calculated using Wyatt DAWN^®^ HELEOS^®^ II MALS, using a dn/dc of 0.185. The resulting chromatograms were analyzed using ASTRA^®^ software, V.6.1.2.84 (Wyatt Tech Corp). The analyses used three conditions, as described in results. For + ATP-Mg^2+^ reactions, standard running buffer was supplemented with 0.2 mM ATP and 0.2 mM MgCl_2_ and HelQ was pre-incubated in 5 mM MgCl_2_ and 5 mM ATP at 37°C for 10 min. For reactions + ssDNA, HelQ was pre-incubated in standard buffer + ATP-Mg^2+^ and with the addition of 5 μM of ssDNA that was strand-1 from fork-2, detailed in Supplementary Figure S1.

### Measurements of DNA fluorescence anisotropy

Protein–DNA interactions were detected using the PerkinElmer EnVision benchtop plate reader (2105). Software used was Wallac EnVision Manager using the FP Fluorescein Dual filters. The software converted fluorescence emission values into mP (fluorescent polarization) values using the equation mP = 1000 x (S-GXP)/(S+GXP); Where S and P are the emission filters and G is a factor to correct for effect of the emission filter transmission variations. Reactions were set up with 1×HB, 25 mM DTT, fluorescein labelled fork DNA (to optimized concentration) and loaded into a 384 Nunc black plate. HelQ protein was diluted to concentrations indicated in results and added to the reaction plate to a final volume of 50 μl. Readings were taken at 0, 5, 10 and 15 min at 30°C, calibrated with a zero-protein control, and plotted as change in mP against protein concentration using PRISM.

### BamHI^EIIIA^ helicase assays

Helicase activity of HelQ in the presence of BamHI^EIIIA^ were carried out as for standard helicase assays, but with pre-incubation of BamHI-EIIIA with the DNA fork at described concentrations at ambient temperature for 15 min prior to addition of reaction components and HelQ protein.

### RPA-HelQ protein pull-down assays

Protein pull down assays between HelQ or N-HelQ and RPA were carried out using streptactin resin to trap (His)_6_-strep-tagged N-HelQ/HelQ in a gravity flow 0.2 ml streptactin-sepharose column (IBA). HelQ and RPA were pre-incubated in a 1:1 molar ratio on ice for 30 min in buffer comprising 50 mM Tris pH7.5, 10% v/v glycerol, 25 mM DTT, 150 mM NaCl and 0.02% v/v Tween 20. Proteins were applied to streptactin columns equilibrated in the same buffer at 4°C, with flow-through samples being collected and re-applied to the column three times for maximal HelQ binding. Columns were then washed with three column volumes of equilibration buffer prior to elution of bound HelQ in three column volumes using the same buffer containing 2.5 mM desthiobiotin. Collected samples were analysed by SDS-PAGE 8% w/v acrylamide gels.

### Analysis of N-HelQ sequence and structure homologies

The amino acid sequence of N-HelQ was confirmed by mass spectrometry and used to model the tertiary structure of this region in PHYRE2 (43). Resulting predicted structures were analysed and superimposed with DALI (44) giving a structural homology match for Brr2 PWI from PDB accession: 5DCA. The amino acid sequences corresponding to the four-helix bundle PWI domain were aligned for multiple HelQ orthologues and Brr2 helicases using Clustal Omega.

## RESULTS

### Helicase-active HelQ dimers do not require a large HelQ N-terminal region

**Figure 1.**
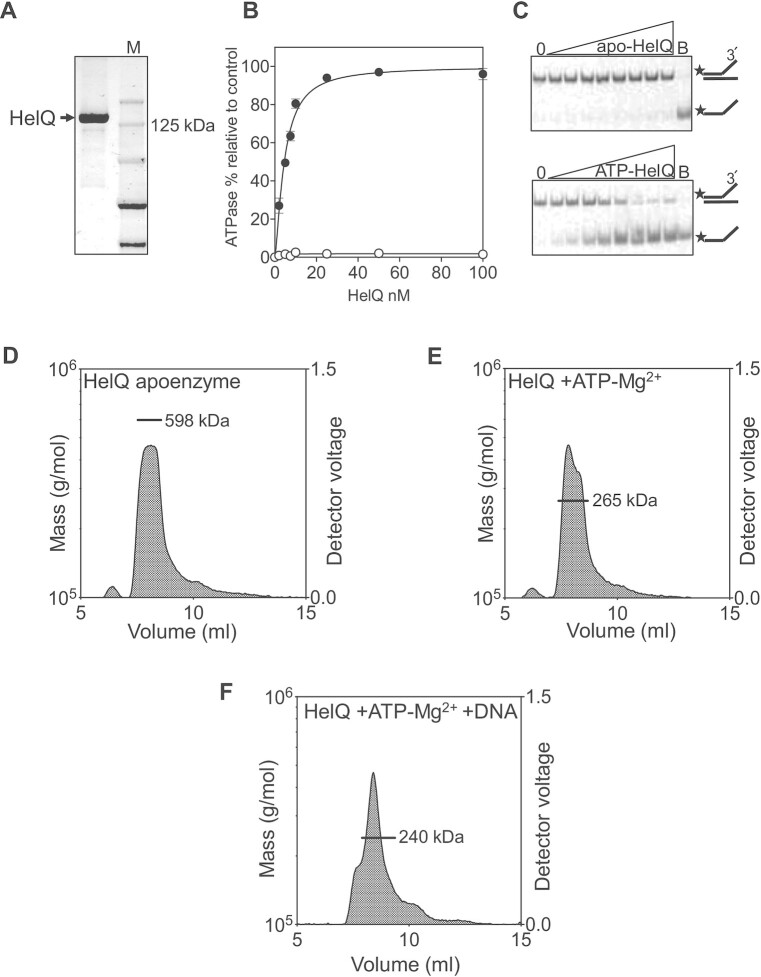
HelQ apoenzyme is activated into a dimer by ATP-Mg^2+^ and ssDNA. (**A**) Purified His-SUMO-SF-tagged HelQ used in this work (calculated molecular mass of 141 kDa) shown coomassie stained after 8% acrylamide SDS-PAGE. (**B**) HelQ (0–100 nM) ATPase activity in the absence of ssDNA (white circles) and when 25 nM was added (black circles). Reactions were in duplicate, and standard error from the mean is shown. ATPase activity is reported relative to a no protein control (‘blank’) reaction. (**C**) HelQ (0–100 nM) helicase activity unwinds fork-2 substrate dependent on 5 mM ATP-Mg^2+^. Stars indicate position of the Cy5-DNA end label, and B indicates a boiled reaction to fully dissociate all DNA duplex. (**D**–**F**) SEC-MALS measurements of HelQ apoenzyme (598 kDa), compared with activated HelQ in buffer containing 5 mM ATP and 5 mM Mg^2+^ (265 kDa), or ATP-Mg^2+^ plus 25 nM of ssDNA (240 kDa).

**Figure 2.**
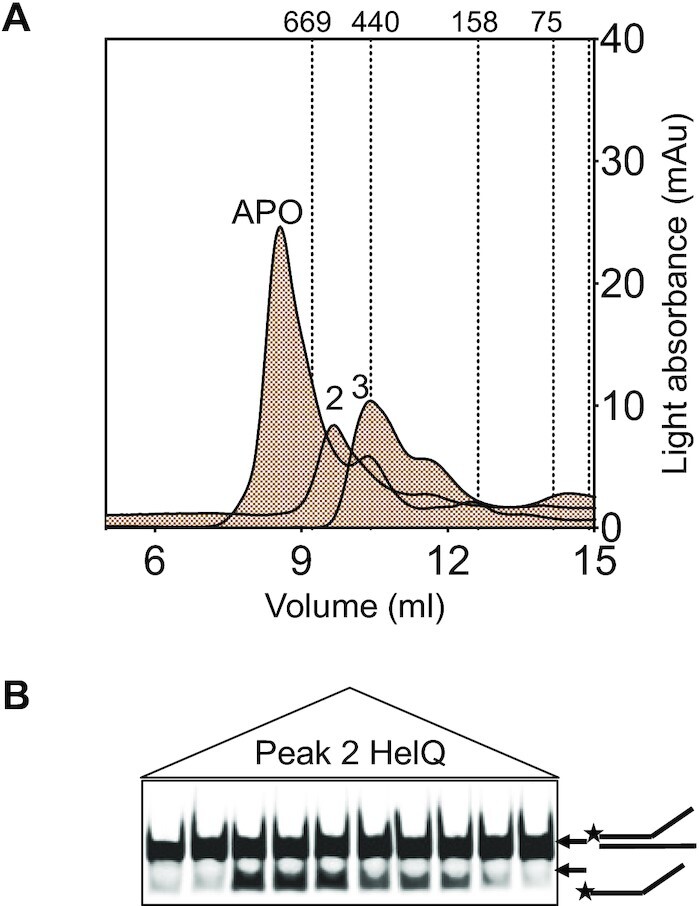
AGF analysis of HelQ proteins. (**A**) apoenzyme (peak APO) compared with HelQ in ATP-Mg^2+^ (peak 2) and in ATP-Mg-ssDNA (peak 3), showing progressively slower migration of HelQ through a Superdex-200 column. Also indicated are the elution volumes for proteins of known mass as indicated. (**B**) HelQ-containing fractions from AGF peak 2 (+ATP-Mg^2+^) unwound fork-2 DNA without addition of more ATP-Mg^2+^ to the reactions, indicating that this is active HelQ.

The active state of HelQ when translocating DNA is not presently known, therefore we compared quaternary structures of purified apoenzyme human HelQ (Figure 1A, a 141 kDa monomer, including affinity tags) with active HelQ that is proficient at unwinding a forked DNA substrate (‘Fork-2’) through ssDNA dependent ATPase activity (Figure 1B and C). As expected, helicase activity was inactivated by disruption of the HelQ Walker B motif (HelQ^D463A^, Supplementary Figure S4). The HelQ apoenzyme protein mass was 598 kDa when measured by SEC-MALS (Figure 1D), consistent with 600-kDa HelQ apoenzyme described previously (25), indicating tetrameric HelQ. However, addition of ATP-Mg^2+^ resulted in HelQ mass being reduced to 265 kDa in SEC-MALS (Figure 1E), and to 240 kDa on further addition of ssDNA (Figure 1F), more consistent with HelQ dimers. A similar effect of ATP-Mg^2+^ on HelQ was apparent using analytical gel filtration (AGF)—the HelQ apoenzyme eluted close to the column void volume (>669 kDa, peak APO), but its elution volume was substantially increased when ATP-Mg^2+^ was included in running buffer (peak 2), and further when ssDNA was also added (peak 3) (Figure 2A), indicating a much reduced Stokes radius in the presence of ATP-Mg^2+^, in agreement with a substantial decrease in the HelQ protein mass measured *via* SEC-MALS. The HelQ peak 2 fractions containing ATP-Mg^2+^ unwound fork-2 without addition of further ATP-Mg^2+^, confirming that HelQ is catalytically active in this form (Figure 2B).

**Figure 3.**
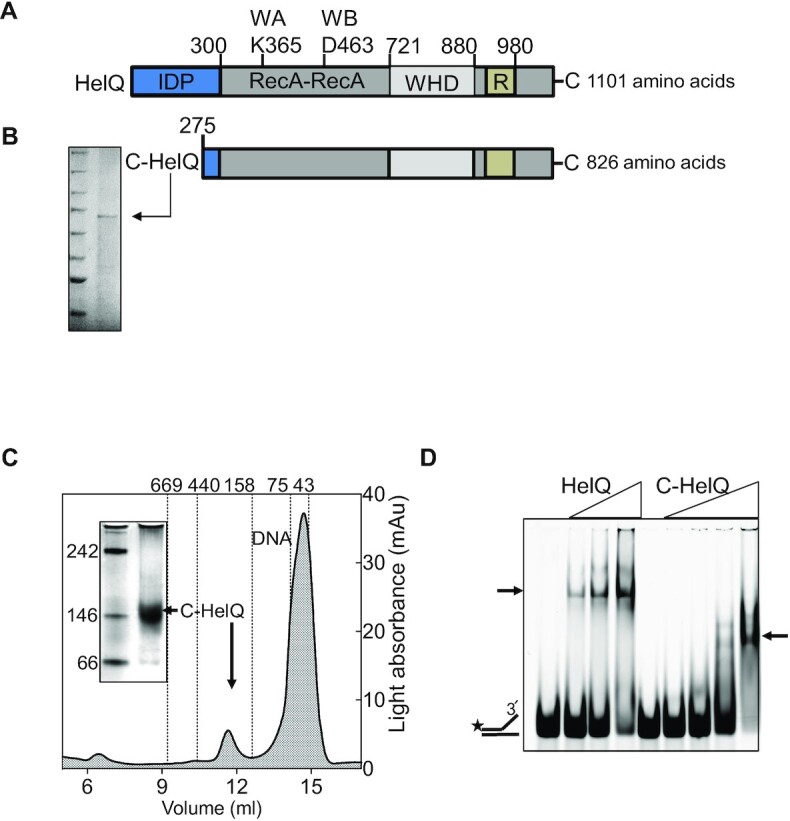
HelQ is catalytically active without the 250–300 amino acid N-terminal region. (**A**) HelQ domains are indicated. The 300-amino acid human HelQ N-terminal region is predicted to be a ‘intrinsically disordered protein’ (IDP); the ‘core’ (C-HelQ) helicase (826 amino acids) comprises RecA-like domains, a winged helix domain (WHD), and a helicase ‘ratchet’ (R). Positions of ATP binding (WA) and ATP hydrolysis (WB) active sites are also indicated. (**B**) Shows purified C-HelQ that (**C**) in AGF and native PAGE of C-HelQ in ATP-Mg^2+^ gave a single population of protein that was consistent with dimer or trimeric C-HelQ. Also indicated are the AGF elution volumes for proteins of known mass as indicated. (**D**) Purified C-HelQ bound to fork-2 DNA (25 nM) to form a stable complex in EMSAs, shown compared with HelQ (both at 40, 80 and 160 nM).

We next investigated if we could isolate a HelQ ‘core’ protein (C-HelQ), comprising only helicase domains, to compare with full length HelQ, aimed at identifying regions of HelQ that are needed for its various functions. To generate C-HelQ we used the 826 amino acids that matched most strongly with the closest homologue of HelQ, the euryarchaeal Ski2 helicase Hel308 (25–30% amino acid identity) (Figure 3A and Supplementary Figure S5). This therefore excluded the 274 amino acid N-terminal region of HelQ that lacks sequence homology to other proteins (Figure 3A) – properties of that region of HelQ are presented later. We purified C-HelQ of the correct size (85 kDa) and its identity was confirmed by mass spectrometry (Figure 3B and Supplementary Table S1). C-HelQ formed a single species in BN-PAGE that migrated close to the 146 kDa marker (Figure 3C), and in AGF a single C-HelQ peak was detected in ATP-Mg^2+^ and ssDNA that sized to ∼228 KDa (Figure 3C and Supplementary Table S2), compatible with C-HelQ dimers that bound to fork-2 DNA in EMSAs (Figure 3D).

**Figure 4.**
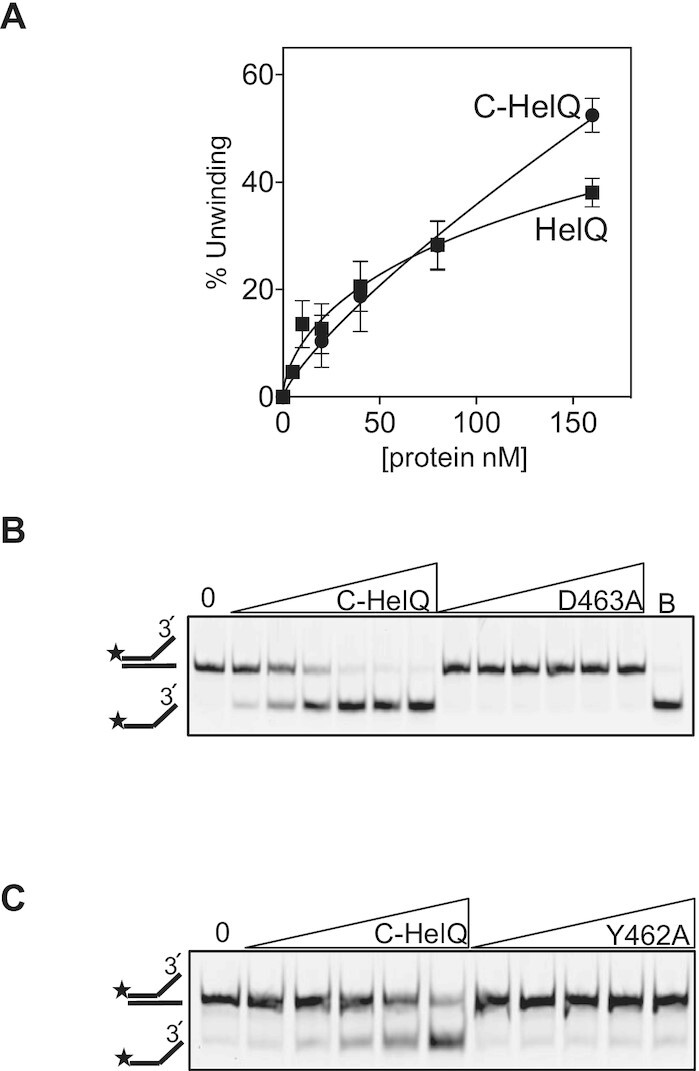
C-HelQ is a functioning helicase core enzyme. (**A**) C-HelQ catalysed unwinding of fork-2 DNA (25 nM) comparably to full length HelQ (both used at 5, 10, 20, 40, 80 and 160 nM). Reactions were in triplicate, and standard error from the mean is shown in bars. (**B**) Helicase unwinding of fork-2 (25 nM) by C-HelQ (0, 20, 40, 60, 120 and 240 nM, B is a boiled reaction) was inactivated by disruption of the ATPase (WB) active site (D463A), and (**C**) C-HelQ (0, 5, 10, 20, 40 and 80 nM) was also inactivated by the amino acid change Y642A in a motif conserved in Ski2 helicases that is needed for DNA translocation but not ATP hydrolysis.

C-HelQ unwound fork-2 similarly to wild-type protein (Figure 4A). As expected, C-HelQ helicase activity was inactivated by the D463A ATPase active site substitution used for full-length HelQ (Figure 4B), and the amino acid substitution Y642A also inactivated C-HelQ (Figure 4C)—this is located in helicase motif IV that is thought to facilitate protein conformational flexibility necessary for DNA translocation in some families of helicases (45). Inactivity of C-HelQ^Y642A^ suggests a similar mode of action for DNA translocation by HelQ. We conclude that the N-terminal region of HelQ is not required for DNA helicase activity, and that HelQ activated by ATP forms active dimers. Attempts to trap ATP-induced functional HelQ dimers by chemical crosslinking were ineffective—the resulting HelQ protein was catalytically inactive and unable to bind to DNA (Supplementary Figure S6).

### HelQ helicase activity is blocked by a single abasic site, G4 DNA and protein barriers

**Figure 5.**
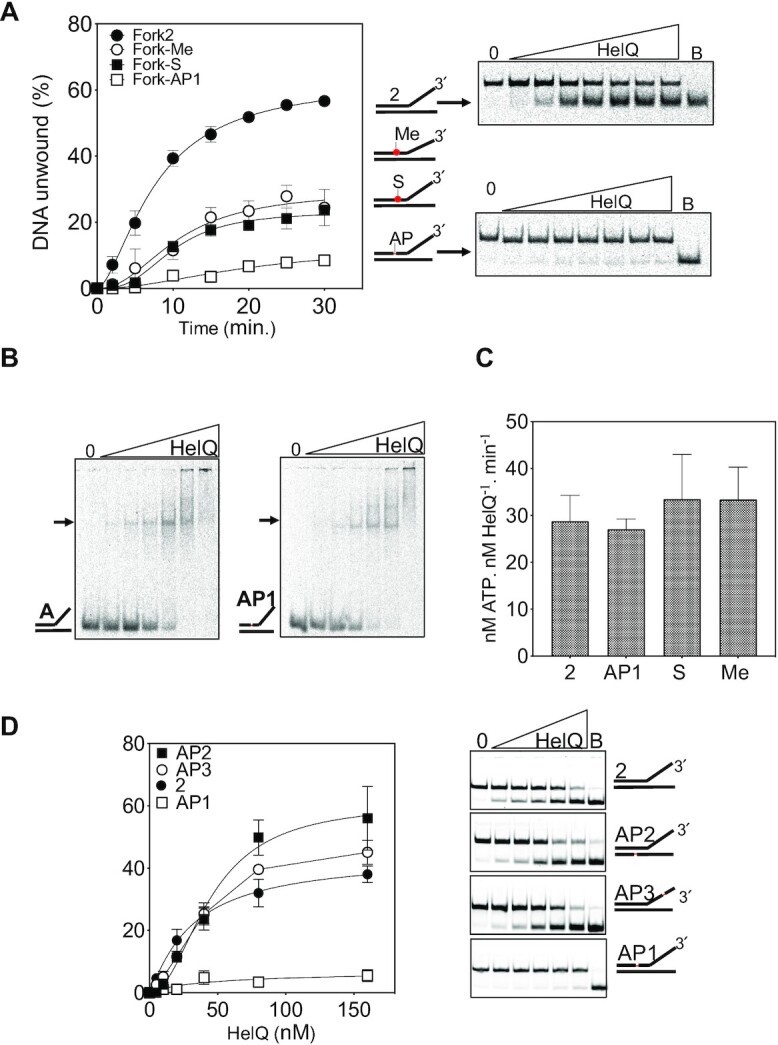
Insights into the translocation mechanism of HelQ. (**A**) DNA unwinding by HelQ was compared for unmodified fork-2 and fork-2 containing chemical modification at a single site, indicated to the right of the graph; an abasic site (AP1), a methyl phosphonate (CH_3_) and a phosphorothioate (S). Also shown are representative TBE gels for HelQ unwinding fork-2 compared with fork-AP1, the most effective inhibitor of HelQ. HelQ was used at 0, 5, 10, 20, 40, 80 and 160 nM, and B denotes a boiled reaction to fully dissociate the fork. (**B**) Representative gels showing that HelQ (0–160 nM) bound to fork-AP1 similarly to fork-2 in EMSAs, and (**C**) that the ATPase activity of HelQ at 80 nM was not disrupted when mixed with each of the modified forks as indicated (25 nM). (**D**) Fork-2 was further modified with AP-sites in the locations indicated and these were used to measure helicase activity of HelQ. AP-DNA was inhibitory on when present within the duplex region translocating strand. Reactions were in duplicate, and standard error from the mean is shown. The panels are from gels showing representative unwinding of the DNA forks (25 nM) that were used to generate the graph. HelQ was used at 10, 20, 40, 80 and 160 nM. The lane labelled ‘B’ is of a boiled reaction to fully dissociate the fork DNA to ssDNA.

We next introduced chemical modifications into fork-2 at single sites and assessed their effects on HelQ and C-HelQ helicase activity—positions of the chemical modifications and their chemical structures are detailed in Supplementary Figure S1. These were designed to provide information about how HelQ interacts with the overall fork-2 structure and its DNA backbone and bases. HelQ (160 nM) unwound 60% of fork-2 (25 nM) after 30 min, which was reduced to 7% when a single abasic site was located five nucleotides into the duplex region of the 3′ to 5′ translocating strand (Figure 5A, fork AP1). To ensure that this fork AP1 can be catalytically unwound we used *E. coli* RecQ helicase to fully dissociate it without inhibition from the abasic site (Supplementary Figure S7A). A single methyl-phosphonate or phosphonothioate DNA backbone modification at the same position was less inhibitory, resulting in 20–25% of fork being unwound by HelQ (Fork-Me and Fork-S, Figure 5A). HelQ DNA binding was unaffected by any of the chemical modifications, summarized for Fork-AP1 that showed the most severely reduced helicase activity (Figure 5B). HelQ hydrolysed ATP effectively even when its helicase activity was severely inhibited by the abasic site (Figure 5C), a functional uncoupling observed for other helicases from diverse families, including RecBC (46), PcrA (47), Has1p (48) and BLM (49). These data indicate that inhibition of HelQ by DNA modifications was specific for translocation activity and was not caused by changes in DNA binding and/or ATPase activities.

**Figure 6.**
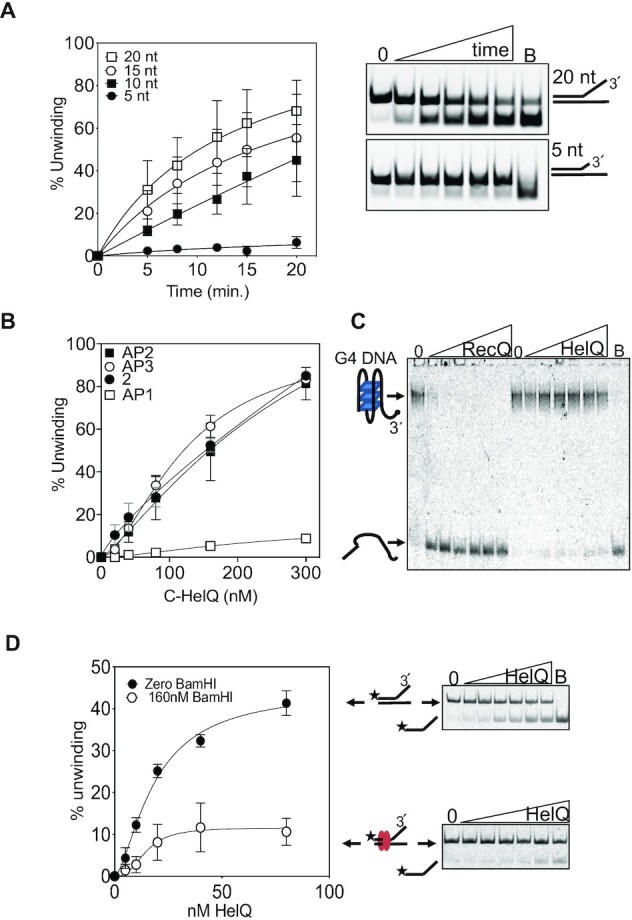
(**A**) Effect on HelQ helicase activity of varying the length of available 3′ ssDNA at the branchpoint of fork-2 substrate. The graph shows HelQ (50 nM) unwinding the substrates (25 nM) as a function of time (0–20 min.), in duplicated reactions with standard error from the mean indicated. Two representative assays are shown as gel panels next to the graph, lanes marked ‘B’ indicate boiled reaction mixtures. (**B**) Inhibition of C-HelQ (0–300 nM) helicase activity followed the same pattern as for full-length HelQ when mixed with the DNA forks AP1, AP2 and AP3. Reactions were in triplicate, and standard error from the mean is shown. (**C**) G4 quadruplex DNA inhibited HelQ (0–160 nM) helicase activity compared with *Escherichia coli* RecQ (0–160 nM) used as a positive control. Lane ‘B’ indicates G4 DNA that was boiled to fully dissociate DNA strands. (**D**) Fork DNA (25 nM) that was 100% bound by BamHI^E111A^ (160 nM, see also Supplementary Figures S4 and 7) was unwound by HelQ (0–160 nM, white circles) less effectively than unbound fork (black circles). Representative gels are shown of reactions that were repeated three times, with standard error from the mean shown.

The strong inhibitory effect of the abasic site was used to examine further how HelQ engages to unwind the fork-2 DNA. For this, we used end-point helicase assays titrating HelQ at concentrations from five-fold lower to 10-fold higher than DNA, in which Fork-AP1 was again unwound poorly (5% of DNA unwound) (Figure 5D). Moving the abasic site to the equivalent position in the opposite DNA strand had no inhibitory effect on HelQ (Figures 5D, Fork-AP2), indicating that HelQ translocates on one ‘tracking’ DNA strand. Positioning the abasic site in the tracking strand within ssDNA seven nucleotides away from the fork branchpoint also did not inhibit HelQ (Figures 5D, Fork-AP3). This suggests that HelQ can load onto the DNA fork close to the ssDNA-dsDNA branchpoint and does not translocate across the AP3 abasic site—if it did, we would expect to observe at least partial inhibition of fork unwinding. We assessed this further, in the knowledge that HelQ tracks along one strand of the fork with 3′ to 5′ polarity, by varying the length of 3′ ssDNA available at the branchpoint and measuring helicase unwinding (Figure 6A). As was expected from a previous study (26), HelQ was inactive at unwinding a substrate lacking any 3′ ssDNA at all, it unwound only 5% of DNA with 5 nt of 3′ ssDNA (Fork 2.05, Supplementary Figure S1A) despite binding normally to it (Supplementary Figure S7B), but progressively recovered full unwinding activity (50–60% of DNA unwound) from 10–20 nt of 3′ssDNA (Forks 2.10–2.20). Therefore, the presence of DNA fork branchpoint alone is sufficient for HelQ binding to DNA but is not sufficient for triggering ssDNA stimulated ATPase and translocation activities. Finally, DNA unwinding by the core HelQ helicase, C-HelQ, was also strongly inhibited by Fork-AP1, but not by abasic sites in Fork-AP2 or AP3 (Figure 6B), further evidence that HelQ and C-HelQ have the same helicase mechanism that does not require the 274 amino acid N-terminal region.

We next assessed if HelQ could overcome a range of other potential barriers to DNA translocation. HelQ unwound fork-2 modified into an RNA–DNA hybrid in which the 3′ tail was RNA (Supplementary Figure S7C), indicating the it can translocate RNA as well as DNA. However, HelQ did not unwind a model G4 DNA substrate (40)—four-stranded DNA in cells implicated in genome instability and regulation, reviewed most recently in (50)—that was unwound by bacterial RecQ used as a positive control (Figures 6C). The inability of HelQ to generate force sufficient to dissociate G4 DNA suggested that it may also be unable to disrupt a protein–DNA barrier. To test this, we used catalytically inactive BamHI^E111A^ bound to DNA (*K*_d_ 2.95 × 10^−11^ M (51)) that has been used in previous studies to form a barrier to DNA unwinding (52,53). We incorporated the hexanucleotide BamHI binding recognition site into the duplex region of a DNA fork (Supplementary Figure S1A) and observed that 100% of this fork was bound by using 160 nM of BamHI^E111A^ (Supplementary Figure S8). In the same conditions HelQ helicase activity unwound only 10% of this DNA substrate compared to 50% when BamHI^E111A^ was absent (Figure 6D), indicating that HelQ is ineffective at displacing BamHI^E111A^. We conclude that the DNA unwinding, but not ATP hydrolysis, activity of HelQ is especially sensitive to an abasic site and is unable to displace DNA barriers but is more tolerant of chemical changes to the DNA/RNA backbone.

### A non-catalytic PWI domain in the HelQ N-terminal region destabilizes RPA-DNA binding

**Figure 7.**
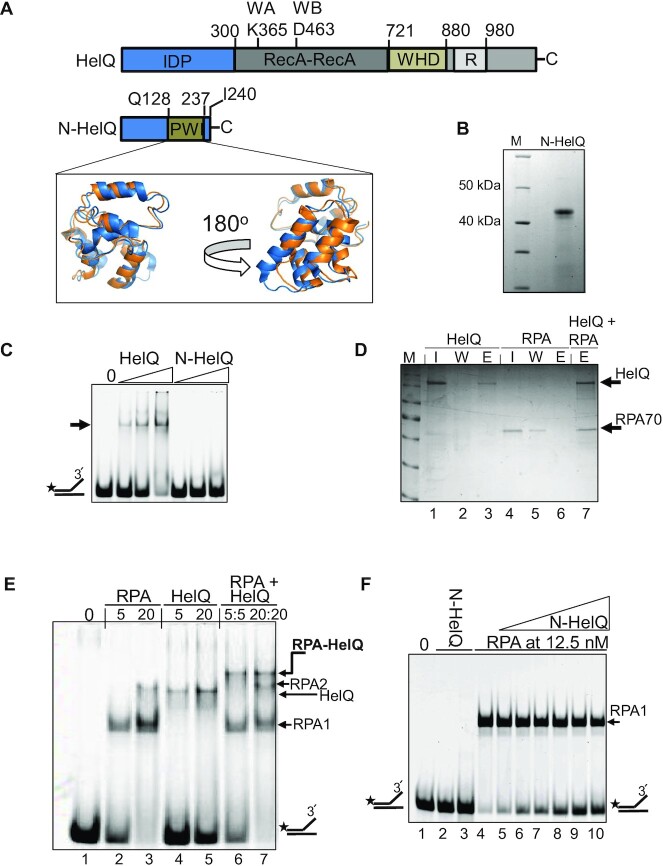
A predicted PWI-like fold in the non-catalytic HelQ N-terminal region destabilizes RPA–DNA complexes. (**A**) The 300 amino acid N-terminal region of human HelQ (N-HelQ) lacks sequence homology with other proteins and is predicted to be an IDP. Searches for predicted structural homology of N-HelQ identified a strong match between N-HelQ residues 128–237 (orange) with a four-helix bundle PWI-fold in crystal structures of RNA helicase Brr2 (blue). (**B**) Purified N-HelQ (47 kDa including a His-SUMO tag) did not bind to fork-2 DNA (25 nM) in EMSAs, (**C**) when used at 0–500 nM compared to HelQ (0–160 nM). (**D**) *In vitro* reconstitution of HelQ-RPA70 physical interaction utilizing streptavidin-tagged HelQ and untagged RPA70, in the absence of DNA. HelQ input (I, 20 μg, lane 1) that bound to affinity beads was eluted (**E**) using desthiobiotin buffer (lane 3) after washing with PBS (W, lane 2). RPA70 alone (I, 20 μg, lane 4) did not bind to the affinity beads (lanes 4–6) but co-eluted with HelQ that was pre-bound to the beads (lane 7). (E) Evidence for physical interaction of HelQ with pre-bound heterotrimeric RPA–DNA complex in EMSAs. Fork-2 DNA (25 nM) was bound by RPA or HelQ (each at either 5 or 20 nM) as indicated, or HelQ was added to pre-bound RPA–DNA complex as indicated, generating a super-shifted RPA–HelQ complex (lanes 6 and 7). (**F**) RPA (12 .5 nM) pre-bound 100% of fork-2 (25 nM, lane 3), that was reduced on addition of N-HelQ (0–500 nM) during 10-min reactions and a further 45 min of electrophoresis. N-HelQ (500 nM) did not bind to fork-2 DNA, shown in duplicate in lanes 2 and 3.

A 250–300 amino acid N-terminal region (Figure 7A) of metazoan HelQ proteins is presently of unknown function and is not needed for DNA binding or translocation by HelQ (Figures 3 and 4). This region, which we refer to as N-HelQ, lacks overall sequence homology with other proteins and is predicted to be intrinsically disordered (Supplementary Figure S9). However, searches for potential structural homologies identified a single strong match between N-HelQ amino acids 128–237 and the four-helix bundle of a PWI-fold in the crystal structure of a yeast Ski-2 family helicase Brr2 (Figure 7A and Supplementary Figure S10) (54). To investigate this further, we purified human N-HelQ containing the predicted PWI-fold (amino acids 1–240, 46 kDa including the affinity tags for purification), first as a fragment of full HelQ that arose during its purification that was identified by mass spectrometry (Supplementary Figure S2), and this information was used to clone the corresponding DNA for over-production and purification of N-HelQ (Figure 7B). N-HelQ could be purified in large quantities for analysis by analytical ultra-centrifugation that gave a mean mass of 40.1 KDa over three conditions (Supplementary Figure S11A) consistent with it being a monomer.

The PWI-fold of Brr2 and related proteins interacts with DNA and proteins. N-HelQ did not bind to single-stranded, duplex or forked DNA in EMSAs or in anisotropy measurements compared with a DNA binding control (Figure 7C and Supplementary Figure S11B). We therefore investigated for protein interactions beginning with RPA, which interacts with HelQ protein in human cells—the interaction was observed to be with RPA70, the ssDNA binding subunit of heterotrimeric RPA complex (4,26). We purified RPA heterotrimer (Supplementary Figure S3) and reconstituted physical interaction with HelQ *in vitro*. In the absence of DNA, the RPA70 subunit co-eluted with affinity immobilized HelQ (Figure 7D), and in the presence of DNA in EMSAs a pre-formed RPA–DNA complex (Figure 7E, lanes 2 and 3) was ‘super-shifted’ into a slower migrating complex on addition of HelQ. HelQ did not cause this effect when added to pre-bound *E. coli* SSB-DNA complex (Supplementary Figure S12). Therefore, this established two methods for next testing physical interaction of N-HelQ with RPA *in vitro* when RPA is either bound to DNA or not (Figure 7E, lanes 6 and 7). However, substituting N-HelQ in place of HelQ did not reproduce interaction with RPA in either scenario, or when using alternative and potentially more sensitive methods—see ‘Discussion’ section. Strikingly though, in EMSAs although no super-shift of RPA–DNA was observed the N-HelQ protein instead destabilized RPA–DNA binding, resulting in accumulation of naked DNA, and despite N-HelQ not binding to DNA (Figure 7F).

**Figure 8.**
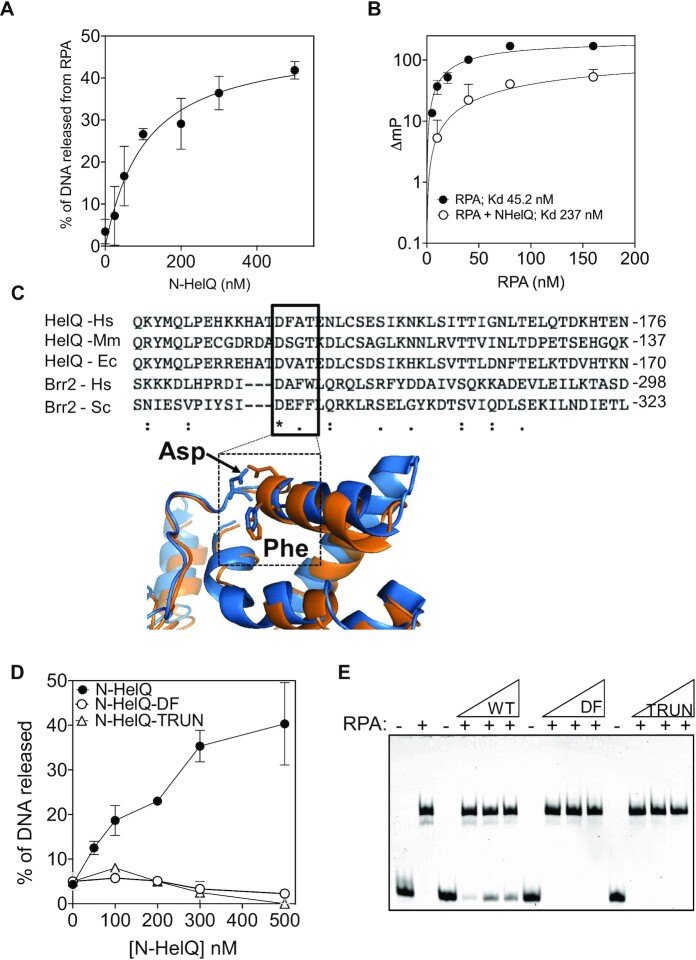
Disruption of the N-HelQ PWI-like fold inactivates RPA displacement. (**A**) N-HelQ dependent displacement of RPA–DNA complex was measured as total free DNA visible, in triplicate in EMSAs with standard error shown. (**B**) Effect of RPA on DNA (fork-2) anisotropy, plotted as change in fluorescent polarization (ΔmP) when mixed with BSA (500 nM, black circles) or N-HelQ (500 nM, white circles). *K*_d_ constants were generated in PRISM using non-linear best fit. (**C**) Alignment of N-HelQ and Brr2 PWI regions showing a conserved aspartic acid (Asp-142)—sequences shown are Hs, human; Mm, mouse; Ec, horse; and Sc, yeast *Saccharomyces cerevisiae*). Superimposition of N-HelQ model with yeast Brr2 crystal strcuture (PDB ID: 5DCA) highlighting relative positioning of the Asp-142 and Phe-143 of N-HelQ that were targeted for mutagenesis in N-HelQ. (**D**) Comparison of DNA released from pre-bound RPA–DNA complex measured in EMSAs for N-HelQ (black circles), N-HelQ mutant D142A/F143A (DF, triangles) and truncated N-HelQ lacking the PWI region (TRUN, white circles). Assays were in triplicate data and standard error from the mean values are shown as error bars. (**E**) Summary showing loss of RPA–DNA displacement by mutant N-HelQ proteins as indicated when titrated into RPA (12.5 nM)–DNA complex at 50, 250 and 500 nM.

**Figure 9.**
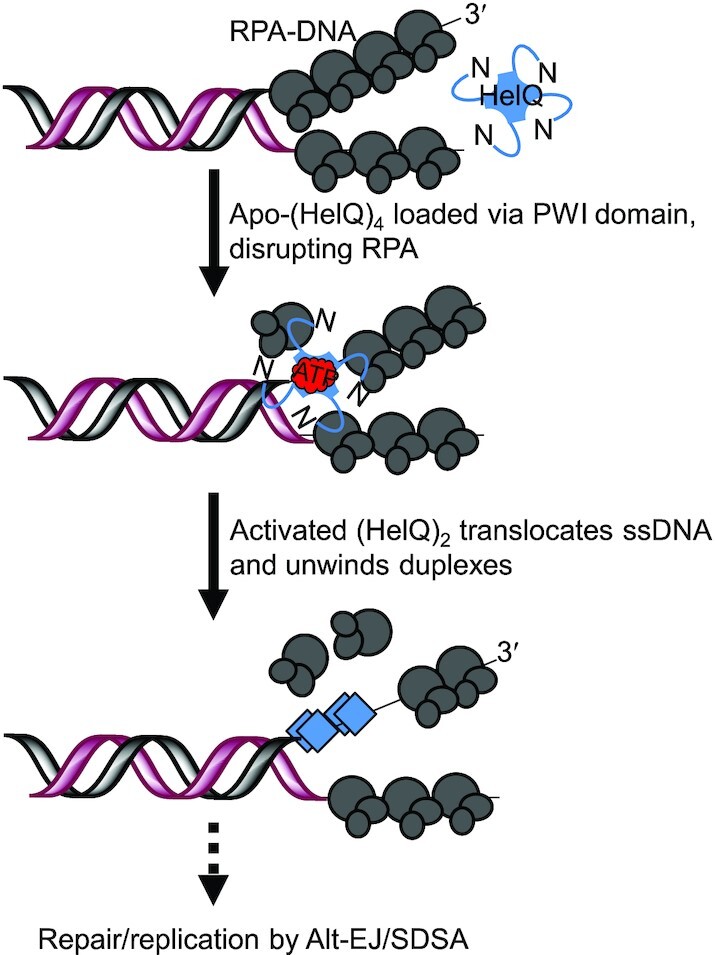
Schematic model for loading and activation of HelQ helicase. HelQ apoenzyme in tetrameric form becomes part of the genome maintenance/DNA repair response to ssDNA arising at stressed forks, where it interacts with RPA through its N-terminal PWI-like domain. This disrupts RPA–DNA complexes sufficiently to accommodate HelQ onto ssDNA—the data suggest that once RPA is evicted from ssDNA by N-HelQ it is unable to immediately re-assemble on to DNA. RPA–DNA binding is conformationally dynamic and these dynamics may be influenced by interacting proteins such as HelQ—a recent review article summarizes these ideas (57). Access to ssDNA triggers HelQ ATPase activity of the core helicase domains in activated dimers. Translocation of HelQ occurs along one DNA strand with 3′ to 5′ directionality. HelQ helicase activity may separate DNA strands to assist with priming replication restart and may influence homologous recombination through physical interactions it forms with Rad51 paralogue complexes.

Measurements of this effect showed that although RPA alone bound 100% of the DNA, 40% of the bound DNA was liberated as free, unbound DNA on incremental addition of N-HelQ (Figure 8A). Further evidence that N-HelQ destabilized RPA–DNA binding was gained by measuring the anisotropy of fork-2 DNA labelled with fluorescein. RPA binding to fork-2 gave a dissociation constant (*K*_d_) of 45.21 nM, which increased to 237 nM on addition of N-HelQ (Figure 8B), and we have already shown that N-HelQ alone did not bind to DNA (Figures 7C  Supplementary and S11B). N-HelQ had no effect on DNA binding by *E. coli* SSB (Supplementary Figure S13). These data suggest that N-HelQ triggers displacement of RPA from DNA, by a mechanism independent of DNA binding by HelQ, which is therefore likely to be through direct interaction with RPA. Because the PWI fold of other Ski-2 like helicases mediates protein interactions we reasoned that the predicted N-HelQ PWI region of N-HelQ may be required to promote dissociation of the RPA–DNA complexes, and we therefore disrupted it. Despite very low overall sequence conservation between the predicted human N-HelQ PWI fold (residues 128–237) and Brr2 PWI, a conserved aspartic acid residue was identified (Asp-142 in human HelQ) that is reportedly necessary for Brr2 function (Figure 8C) (54). In Brr2 this aspartic acid orientates outward from the core of the PWI fold that is stabilized by a nearby phenylalanine residue, which corresponds to Phe-143 in human HelQ. We therefore purified N-HelQ with glycine substituted for both Asp-142 and Phe-143 (N-HelQ^DF-GG^), and additionally a truncated N-HelQ protein that lacks all predicted PWI residues 128–237 (N-HelQ^TRUN^) (Supplementary Figure S14). Neither protein was able to liberate free DNA from RPA–DNA complex when measured in comparison to N-HelQ (Figure 8D and E). Although we could not detect a stable N-HelQ–RPA interaction, we conclude that the N-terminal region of HelQ functionally interacts with RPA bound to ssDNA to displace it, and that this requires a predicted PWI-fold. This activity of N-HelQ suggests a molecular basis for RPA-stimulated HelQ helicase activity (Figure 9) that is discussed below.

## DISCUSSION

HelQ helicase is part of systems in human cells that guard against genetic changes that accumulate as part of carcinogenesis. HelQ is especially associated with DNA repair at sites where aberrant ssDNA has accumulated as a result of blocked replicative helicase or polymerase complexes. Involvement of HelQ in overcoming DNA damage that blocks replication makes it a potential stand-alone helicase target for improving the efficacy of cancer treatments that rely on replication blocking agents to kill cancer cells. In this study we identify that the human HelQ protein comprises a non-catalytic N-terminal region (N-HelQ) for ssDNA loading via RPA, and a fully active ‘core’ helicase (C-HelQ) that hydrolyses ATP and translocates ssDNA without need for N-HelQ. This in particular enabled us to interrogate the mechanisms for DNA loading and translocation by HelQ, generating a model shown in Figure 9.

HelQ requires ssDNA binding to trigger ATP-dependent DNA translocation. Evidence from EMSAs, DNA anisotropy and targeted mutagenesis showed that N-HelQ disrupts RPA–DNA binding that we propose enables loading of HelQ onto ssDNA. The observed effect of N-HelQ on RPA required Asp-142 and Phe-143, located within a PWI-like fold detected amongst regions of intrinsic protein disorder within N-HelQ. Previous work showed that RPA stimulates HelQ helicase activity, but not by RPA preventing re-annealing of unwound DNA (25,26). Our data are consistent with this—RPA-facilitated loading of HelQ onto ssDNA would be more effective for triggering HelQ helicase activity compared to random collision of HelQ with ssDNA that is free of RPA. Targeting and expulsion of RPA-ssDNA by HelQ may also provide a mechanism in cells for localizing HelQ to ssDNA that arises at stressed replisomes. We observed N-HelQ dependent liberation of naked DNA from RPA binding, which suggests that N-HelQ may not only re-model the disposition of RPA when bound to DNA but may even displace it altogether. N-HelQ does not bind to DNA, and this raises the interesting question; why did we see no evidence for RPA re-associating with DNA after displacement? Instead, we observed progressive accumulation of naked DNA. Therefore N-HelQ seems to prevent RPA from re-establishing efficient DNA binding during the assay. RPA polypeptide subunits and individual DNA binding domains within them engage with DNA dynamically, modulated by post-translational modification and interaction with other proteins (35,55–58). HelQ interacts with the RPA70 subunit of the heterotrimer—we speculate that N-HelQ may disrupt the integrity of the RPA oligomer and/or DNA binding domains sufficiently to prevent re-binding of DNA. This would in effect inhibit RPA activity, preventing it from controlling the pathway of subsequent DNA repair, instead favouring processes requiring HelQ.

Using EMSAs and protein–protein co-elution we were able to reconstitute *in vitro* the physical interaction between full HelQ protein and the RPA70 subunit that had been reported previously in human cells (3,4). However, these same methods and others—chemical crosslinking, microscale thermophoresis and isothermal microcalorimetry (data not shown)—were unsuccessful at detecting physical interaction of N-HelQ with RPA. This may be because additional sites in HelQ located outside of the N-terminal region stabilize interaction with RPA to the extent that they are detectable by those methods. In addition, it is possible that post-translational modifications to N-HelQ may modulate its function in this respect, in particular if targeted to the intrinsic disorder detected within N-HelQ. Nevertheless, the predicted PWI fold in N-HelQ matched closely the PWI domain in the crystal structure of a yeast spliceosomal Ski-2 like helicase Brr2 (54,59). Although PWI domains are most widely associated with nucleic acid binding (60–62), the PWI domain of Brr2, matching N-HelQ, has variations—it forms the canonical four-helix bundle of PWIs but lacks the canonical conserved proline, tryptophan and isoleucine (‘PWI’) residues. The PWI variant of Brr2 does not bind nucleic acids but instead mediates protein-protein interactions (54,59,63), consistent with lack of DNA binding and interaction with RPA protein for N-HelQ presented in this work.

SEC-MALS gave a molecular mass of 250 kDa for helicase active HelQ consistent with a protein dimer, compared to 600 KDa for HelQ apoenzyme that indicates a tetramer. In-line with this, the helicase region of the most closely related human protein to HelQ, Polθ helicase-polymerase fusion (encoded by *polQ*) (64), is tetrameric when crystallized with a non-hydrolyzable nucleotide (65). Overall sequence identity between HelQ and the Polθ helicase is 10.8%, rising to 34.0% identity when amino acids from the Polθ interaction surfaces are aligned with HelQ, appearing to cluster in one particular domain of the HelQ helicase monomer (Supplementary Figure S15). HelQ may therefore be available as inactive tetramers in readiness for rapid deployment as active dimers when required for replication-coupled DNA repair. In this scenario we would predict that activation of HelQ requires disruption or re-arrangement of these ratchet interfaces to transition from tetramer to dimer. HelQ binds as a stable complex to DNA in EMSAs in buffer conditions supporting either apoenzyme HelQ (e.g. Figure 3D) or activated dimeric HelQ (Supplementary Figure S16), indicating that DNA binding alone is unlikely to trigger change in oligomeric state. Substantial fork unwinding was observed from a 10 nt 3′ ssDNA overhang but was inactivated by a 5-nt overhang, and HelQ was not inhibited by positioning of an AP site 7 nt distant from the fork branchpoint (Figure 5). This indicates the requirement for loading of HelQ onto at least 6 nt of ssDNA to be effective as a DNA helicase and suggests that once loaded translocation is engaged from close to the branchpoint of this substrate.

HelQ was ineffective at unwinding DNA through physical barriers or by skipping over a single abasic site in the DNA tracking strand (Figure 5). Addition of RPA to these reactions did not improve HelQ DNA unwinding activity, either if RPA was pre-incubated with the DNA or not (Supplementary Figure S17), data that together indicate that HelQ it is unlikely to function as a ‘fork clearance’ protein, unlike other helicases that push through these barriers (52,66–67). Interaction of HelQ with Rad51 paralogue complexes BCDX2 and CX3 (3,4), its disruption of Rad51 bound to duplex DNA (12) and the lack of change to crossover recombination frequency when HelQ is lost (4) suggest that HelQ may be part of the homology-dependent DNA repair systems functioning *via* alternative end-joining (MMEJ/alt-EJ) or DNA annealing (e.g. SDSA) (see reviews (68,69)), as is proposed for Polθ (70,71). Sensitivity of Δ*helq* cells to ICLs, and increased replication stalling in these cells even in the absence of ICLs indicates an important role for HelQ in DNA repair coupled to DNA replication. Proteins of the Fanconi anemia complementation group are key components for overcoming ICLs and other replication blocking lesions in human cells, but HelQ seems to act independently from them. The cellular response to ICLs is an area of great interest, because ICLs are highly toxic biproducts of natural cellular chemistry and they are used in drug therapies against cancerous cells. The protective effect of HelQ against ICLs in healthy cells is also a factor promoting resistance to their use as treatments (21,22), but new knowledge about the molecular mechanisms of HelQ can be developed into ways of overcoming this problem, alongside other emerging helicase targets (72).

## Supplementary Material

zcaa043_Supplemental_Files
