# Supplementary material for: The HelQ human DNA repair helicase utilizes a PWI-like domain for DNA loading through interaction with RPA, triggering DNA unwinding by the HelQ helicase core
Source: NAR Cancer. 2021 Jan 12;3(1):zcaa043. doi: 10.1093/narcan/zcaa043 (PMC8210318; doi:10.1093/narcan/zcaa043)
Supplement: zcaa043_Supplemental_Files [file zcaa043_supplemental_files.zip › NARC - HelQ - Suppl Figs - R1.pdf]

Figure S1

A.

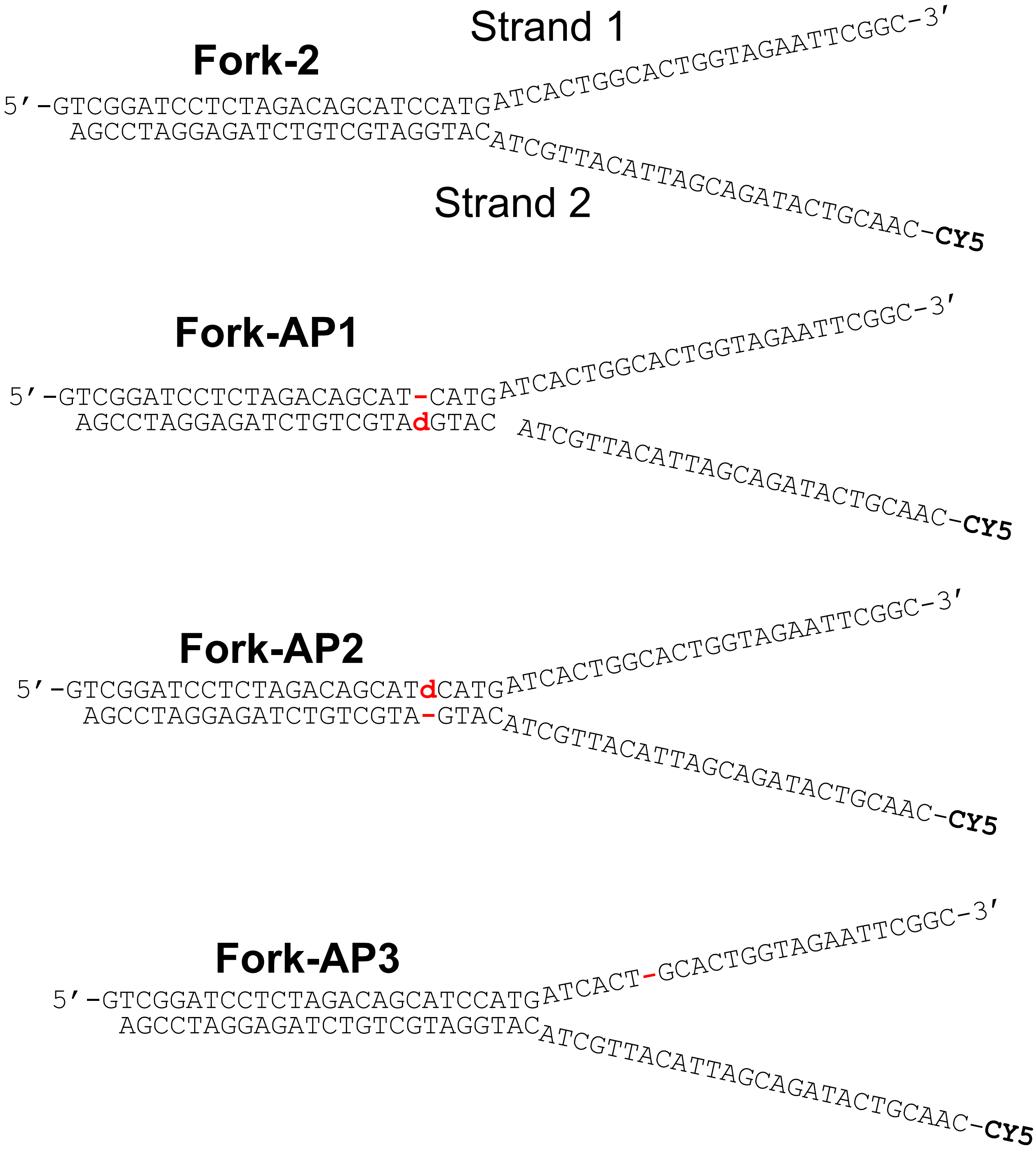

Fork-2
Strand 1
5′-GTCGGATCCTCTAGACAGCATCCATGATCACTGGCACTGGTAGAATTCGGC-3′
AGCCTAGGAGATCTGTCGTAGGTACATCGTTACATTAGCAGATACTGCAAC-CY5
Strand 2
Fork-AP1
5′-GTCGGATCCTCTAGACAGCAT-CATGATCACTGGCACTGGTAGAATTCGGC-3′
AGCCTAGGAGATCTGTCGTAdGTAC ATCGTTACATTAGCAGATACTGCAAC-CY5
Fork-AP2
5′-GTCGGATCCTCTAGACAGCATdCATGATCACTGGCACTGGTAGAATTCGGC-3′
AGCCTAGGAGATCTGTCGTA-GTACATCGTTACATTAGCAGATACTGCAAC-CY5
Fork-AP3
5′-GTCGGATCCTCTAGACAGCATCCATGATCACT-GCACTGGTAGAATTCGGC-3′
AGCCTAGGAGATCTGTCGTAGGTACATCGTTACATTAGCAGATACTGCAAC-CY5

Figure S1

A. (cont.)

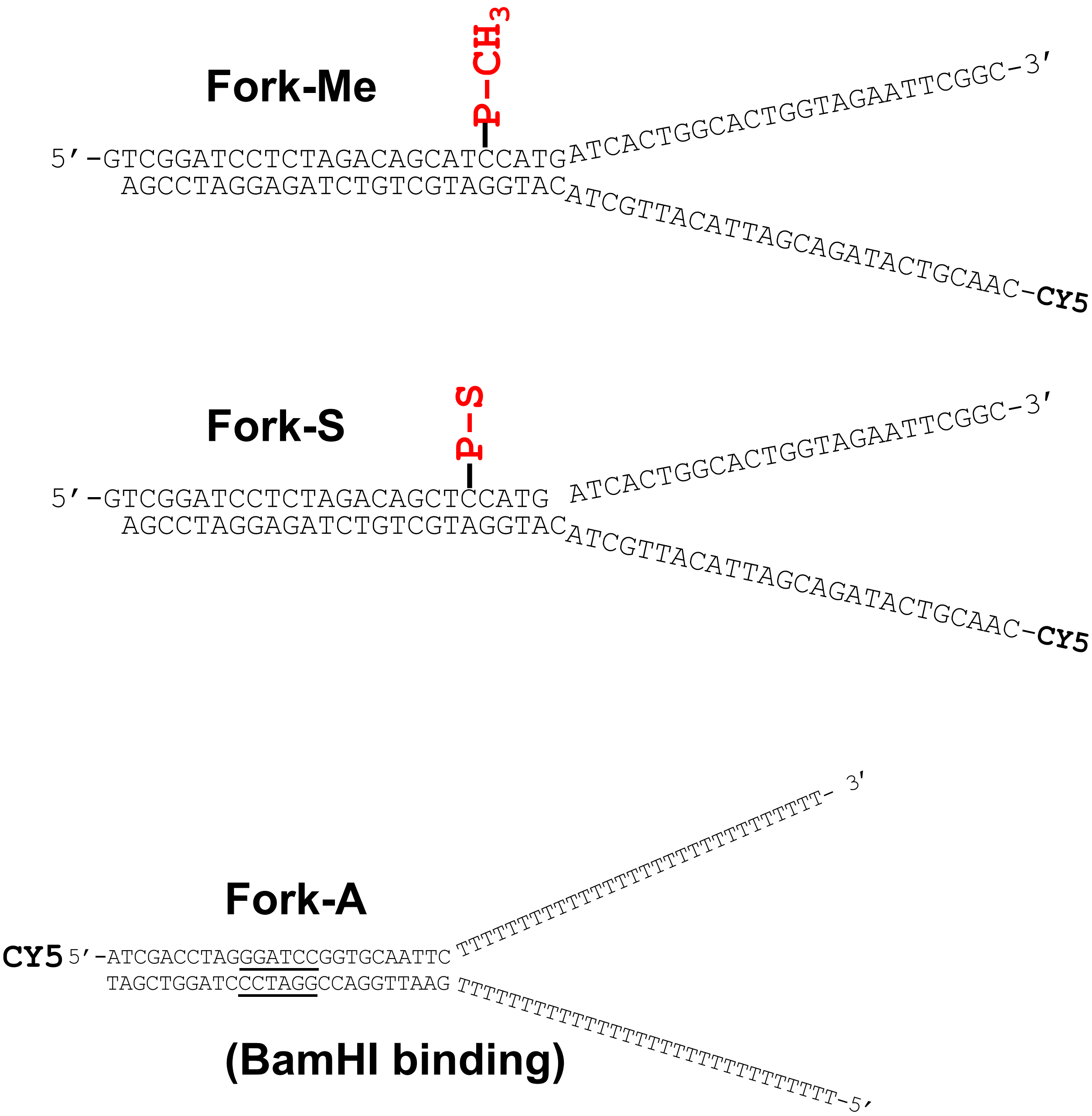

Figure S1

A. (cont.)

Fork-2.00

Strand 00

5′-GTCGGATCCTCTAGACAGCATCCATG-3′
AGCCTAGGAGATCTGTCGTAGGTACATCGTTACATTAGCAGATACTGCAAC-**CY5**

Strand 2

Fork-2.05

5′-GTCGGATCCTCTAGACAGCATCCATGATCAC-3′ Strand 05
AGCCTAGGAGATCTGTCGTAGGTACATCGTTACATTAGCAGATACTGCAAC-**CY5**

Strand 2

Fork-2.10

5′-GTCGGATCCTCTAGACAGCATCCATGATCACTGGCA-3′ Strand 10
AGCCTAGGAGATCTGTCGTAGGTACATCGTTACATTAGCAGATACTGCAAC-**CY5**

Strand 2

Fork-2.15

5′-GTCGGATCCTCTAGACAGCATCCATGATCACTGGCACTGGT-3′ Strand 15
AGCCTAGGAGATCTGTCGTAGGTACATCGTTACATTAGCAGATACTGCAAC-**CY5**

Strand 2

Figure S1

B.

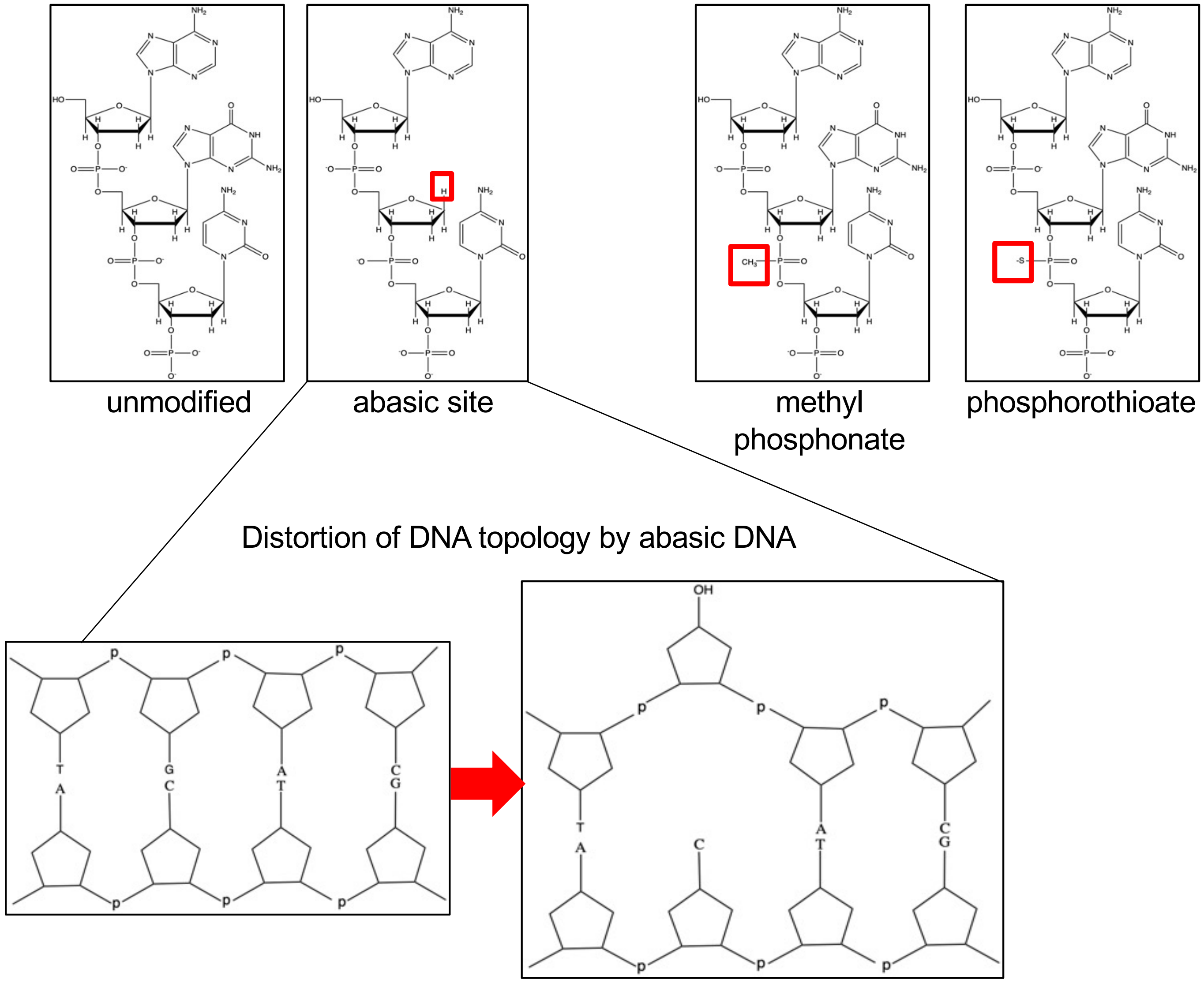

unmodified
abasic site
methyl phosphonate
phosphorothioate
Distortion of DNA topology by abasic DNA

Figure S2

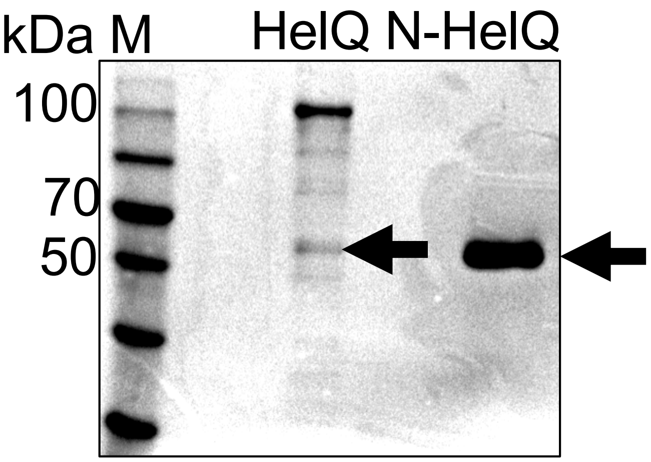

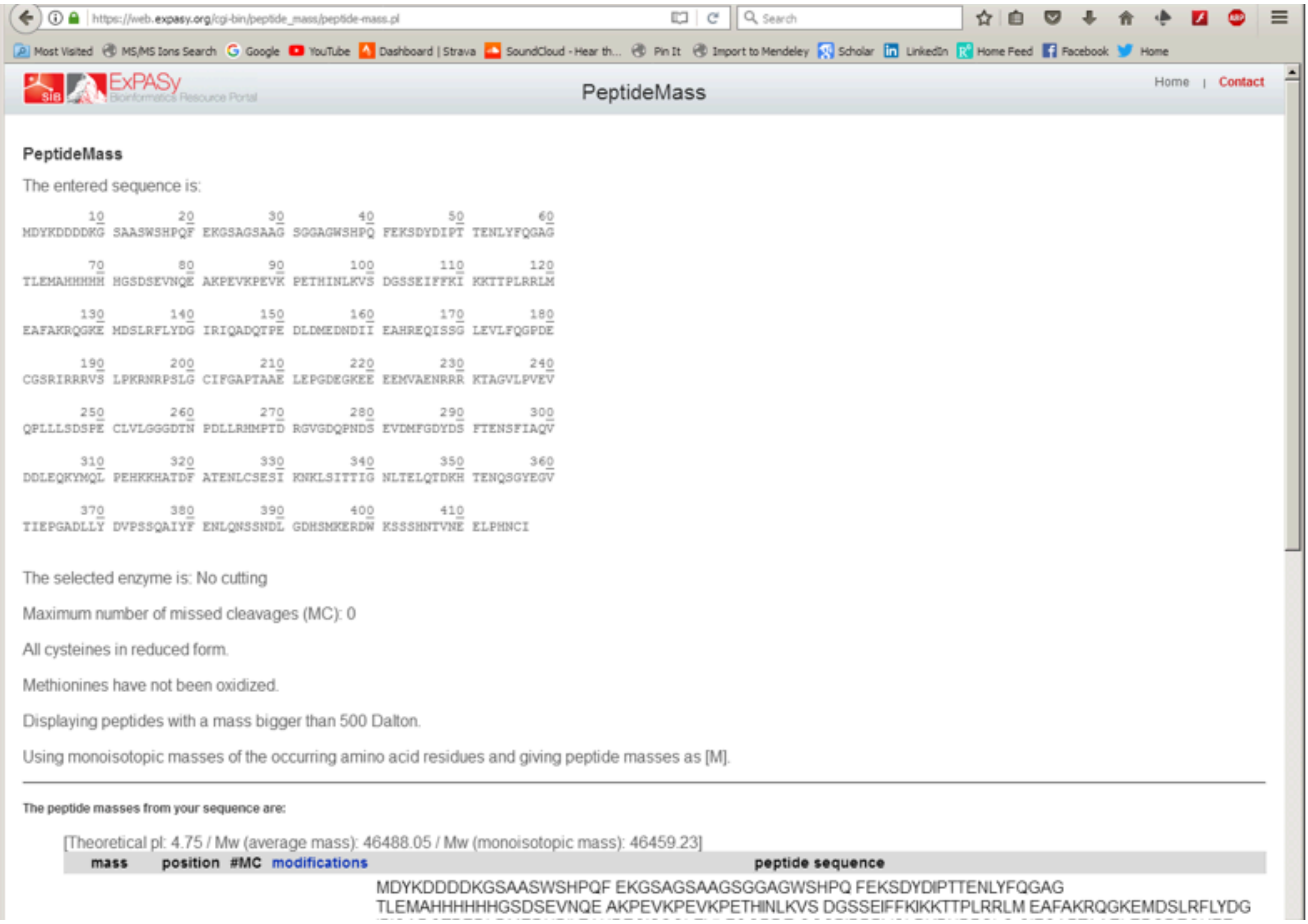

PeptideMass

PeptideMass

The entered sequence is:

```
        10         20         30         40         50         60
MDYKDDDDKG SAASWSHPQF EKGSAGSAAG SGGAGWSHPQ FEKSDYDIPT TENLYFQGAG

        70         80         90        100        110        120
TLEMAHHHHH HGSDSEVNQE AKPEVKPEVK PETHINLKVS DGSSEIFFKI KKTTPLRRLM

       130        140        150        160        170        180
EAFAKRQGKE MDSLRFLYDG IRIQADQTPE DLDMEDNDII EAHREQISSG LEVLFQGPDE

       190        200        210        220        230        240
CGSRIRRRVS LPKRNRPSLG CIFGAPTAAE LEPGDEGKEE EEMVAENRRR KTAGVLPVEV

       250        260        270        280        290        300
QPLLLSDSPE CLVLGGGDTN PDLLRMMPTD RGVGDQPNDS EVDMFGDYDS FTENSFIAQV

       310        320        330        340        350        360
DDLEQKYMQL PEHKKHATDF ATENLCSESI KNKLSITTIG NLTELQTDKH TENQSGYEGV

       370        380        390        400        410
TIEPGADLLY DVPSSQAIYF ENLQNSSNDL GDHSMKERDW KSSSHNTVNE ELPHNCI
```

The selected enzyme is: No cutting

Maximum number of missed cleavages (MC): 0

All cysteines in reduced form.

Methionines have not been oxidized.

Displaying peptides with a mass bigger than 500 Dalton.

Using monoisotopic masses of the occurring amino acid residues and giving peptide masses as [M].

The peptide masses from your sequence are:

[Theoretical pI: 4.75 / Mw (average mass): 46488.05 / Mw (monoisotopic mass): 46459.23]

mass	position	#MC	modifications	peptide sequence
				MDYKDDDDKGSAASWSHPQF EKGSAGSAAGSGGAGWSHPQ FEKSDYDIPTTENLYFQGAG TLEMAHHHHHHGSDSEVNQE AKPEVKPEVKPETHINLKVS DGSSEIFFKIKKTTPLRRLM EAFAKRQGKEMDSLRFLYDG [illegible]

Figure S3

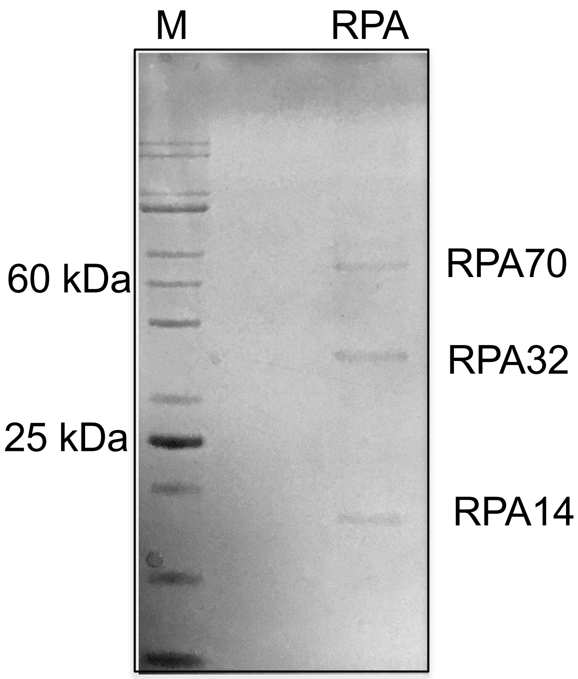

Figure S4

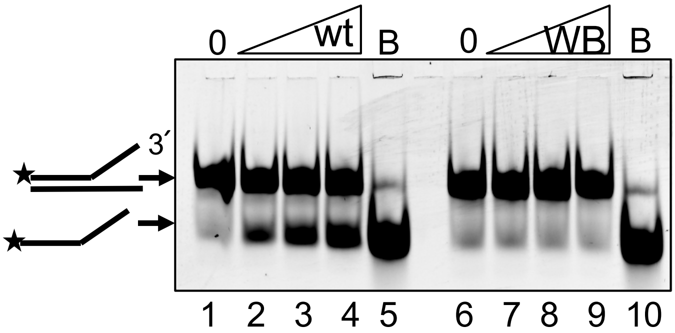

Figure S5

A.

	Description	Max Score	Total Score	Query Cover	E value	Per. Ident	Accession
☑	DEAD/DEAH box helicase [Methanothermobacter tenebrarum]	149	149	41%	3e-37	27.60%	WP_112093728.1
☑	TPA: DEAD/DEAH box helicase [Methanothermobacter sp.]	143	143	41%	2e-35	26.55%	HHW17185.1
☑	DEAD/DEAH box helicase [Methanothermobacter sp. THM-2]	142	142	41%	4e-35	25.52%	WP_160322716.1
☑	DEAD/DEAH box helicase [Methanothermobacter marburgensis]	141	141	41%	1e-34	25.90%	WP_013296018.1

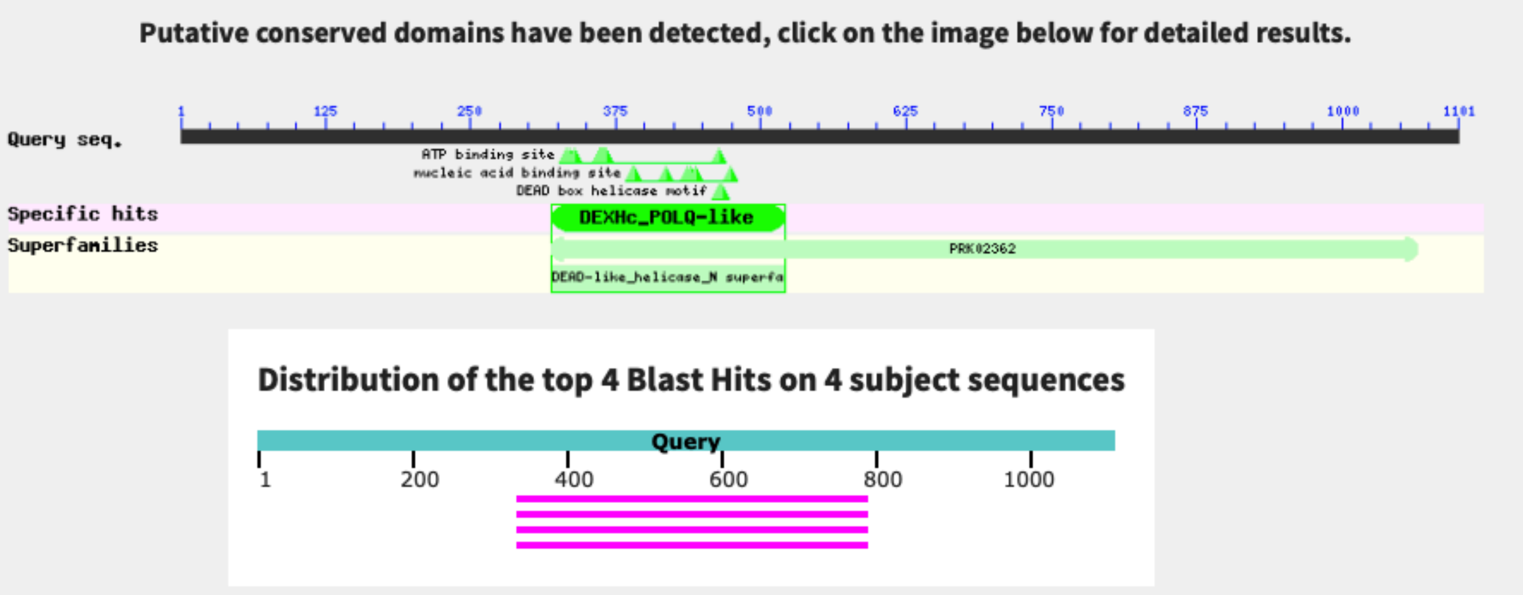

B.

Figure S5

```
  1 MDECGSRIRRRVSLPKRNRPSLGCIFGAPTAAELVPGDEGKEEEEMVAEN     50

  1 --------------------------------------------------      0

 51 RRRKTAGVLPVEVQPLLLSDSPECLVLGGGDTNPDLLRHMPTDRGVGDQP    100

  1 --------------------------------------------------      0

101 NDSEVDMFGDYDSFTENSFIAQVDDLEQKYMQLPEHKKHATDFATENLCS    150

  1 --------------------------------------------------      0

151 ESIKNKLSITTIGNLTELQTDKHTENQSGYEGVTIEPGADLLYDVPSSQA    200

  1 --------------------------------------------------      0

201 IYFENLQNSSNDLGDHSMKERDWKSSSHNTVNEELPHNCIEQPQQNDESS    250

  1 --------------------------------------------------      0

251 SKVRTSSDMNRRKSIKDHLKNAMTGNAKAQTPIFSRSKQLKDTLLSEEIN    300

  1 --------------------------------------------------      0

301 VAKKTVESSSNDLGPFYSLPSKVRDLYAQ-FKGIEKLYEWQHTCLTLNSV    349
                   ..|||.::|.:... :..|.:|...|.:.:....:
  1 ---------------MKSLPPEMRQILGDCYPHIRELNPAQRSAIEAGYL     35

350 QERKNLIYSLPTSGGKTLVAEILMLQELLCCRKDVLMILPYVAIVQEKIS    399
    :...|.|.::||:.||||:..|..|:.::...: |:..:|.::|..|||.
 36 ESEDNYIIAIPTASGKTLLGIIAALKTVMEGGR-VIYTVPLLSIQNEKIK     84

400 GLSSFGIELGFFVEEYAGSKGRFPPTKRREKKSLYIATIEKGHSLV----    445
    ....        :||:....|:.|.|     ..:.:...|...||.
 85 EFRK--------LEEHGIRVGKDPRT-----SDIAVMVFESFDSLTRFSW    121

446 NSLIETGRIDSLGLVVVDELHMIGEGSRGATLEMTLAKILYTSKTTQIIG    495
    |.|.|      :.|::|||.|||||.:||..:|..:.:....:.:.:|:.
122 NILRE------VDLLIVDEFHMIGEYTRGPVIESAITRARTLNPSVRIVA    165

496 MSATLNNVEDLQKFLQAEYYTSQFRPVEL-KEYL--------KINDTIYE    536
    :||||:|::::..:|.|......:|||.| :|.|        :.||.:.:
166 LSATLSNMDEIAGWLDARVVEHDYRPVPLHREVLDTEMFGVREKNDVVLK    215

537 V-DSKAENG-MTFSRLLNYKYSDTLKKMDPDHLVALVTEVIPNYSCLVFC    584
    | :...|:| .|.:.:...:::::|    ..||...::..||:
216 VLERSLEDGSQTLAFVSTRRFTESL----ASHLADKISGKIPD-------    254

585 PSKKNCENVAEMICKFLSKEYLKHKEKEKCEVIKNLKNIGNGNLCPVLKR    634
       ...|:..|:..|.|             ||.|: :.....:.|..|..
255 ---DMVESFREVAGKVL-------------EVPKS-RGSPPTSTCLKLAE    287

635 TIPFGVAYHHSGLTSDERKLLEEAYSTGVLCLFTCTSTLAAGVNLPARRV    684
    .:..|:|:||:||.:.:|:::|:.:..|.:.:.|.|.:|..|||||:|.|
288 CLEAGIAFHHAGLFNRQREIIEDEFRDGNILMITATPSLMYGVNLPSRTV    337
```

B (cont.)

Figure S5

```
 685 ILR--------APYVAKEFLKRNQYKQMIGRAGRAGIDTIGESILILQEK     726
     ::|        .|.....|    .|:||.|||||...|..|.|.||.:..
 338 VIRDYTRWTSQGPRRIPVF----DYEQMSGRAGRPQYDDAGYSYLIARSH     383

 727 DKQQVLE--LITKPLENCYSHLVQEFTKGIQTLFLSLIGLKIATNL----     770
     |:...||  .|...:|...|.:::    ....|:..:|. ::|:.|
 384 DEAMDLEEYYIRGEVERTTSRIIE----NRDALYRQIIA-QVASGLSGTT     428

 771 DDIYHFMNGTFFGVQQKVLLKEKSLWEITVESLRYLTEKG---LLQKDTI     817
     :::..|...||:|.|   :::........::|::|..|..   |::...:
 429 EELADFFRNTFYGYQ---MVEGPFSDSFGMDSIQYEVENATEYLMRNRIL     475

 818 YKSEEEVQYNFHITKLGRASFKG--TIDLAYCDILYRDLKKGLEGLVLES     865
     |...|    .|..|:.|....|.  :::.|   |........::.:.:..
 476 YPGPE----GFSATEFGLLIAKSNYSVETA---IKLHQFASEMDEMDIYR     518

 866 LLHLIYLTTPYDLVSQCNPDWMIYFRQFSQLSPAEQNVAAILGVSESFIG     915
     |::.|..|....|:                                ||.|
 519 LIYEITRTPDMPLI--------------------------------SFKG     536

 916 KKASGQAIGKKVD-----KNVVNRLYLSFVLYTLLKETNIWTVSEKFNMP     960
     :|:......|.::     .:|.|....:..|...:.|...:.:...|::
 537 RKSRDPVRDKLMEHGLFLMDVGNEEATAAALIEWINERTEYEIENAFHV-     585

 961 RGYIQNLLTGTASFSSCVLHFCEELEEFWVYR--ALLVELTKKLTYCVKA    1008
       |..:........|..|..|.:..|...|||  :.|..|:.:|.|.||.
 586 --YAASTRRSAYEASKIVKFFGKICEIMGVYRHSSQLEILSARLYYGVKE     633

1009 ELIPL-MEVTGVLEGRAKQLYSAGYKSLMHLANANPEVLVRTIDHLSRRQ    1057
     :.||| :.|.|:...||::......:.|.|:.    |..::.||.:..:.
 634 DAIPLVVGVRGLGRVRARKIIKTFGEDLRHVR----EDELKRIDGIGPKM     679

1058 AKQIVSSAKMLLHEKAEALQEEVEELLRLPSDFPGAVASSTDKA   1101
     |..|....:..
 680 AGAIRRYCERF---------------------------------    690
```

Figure S6

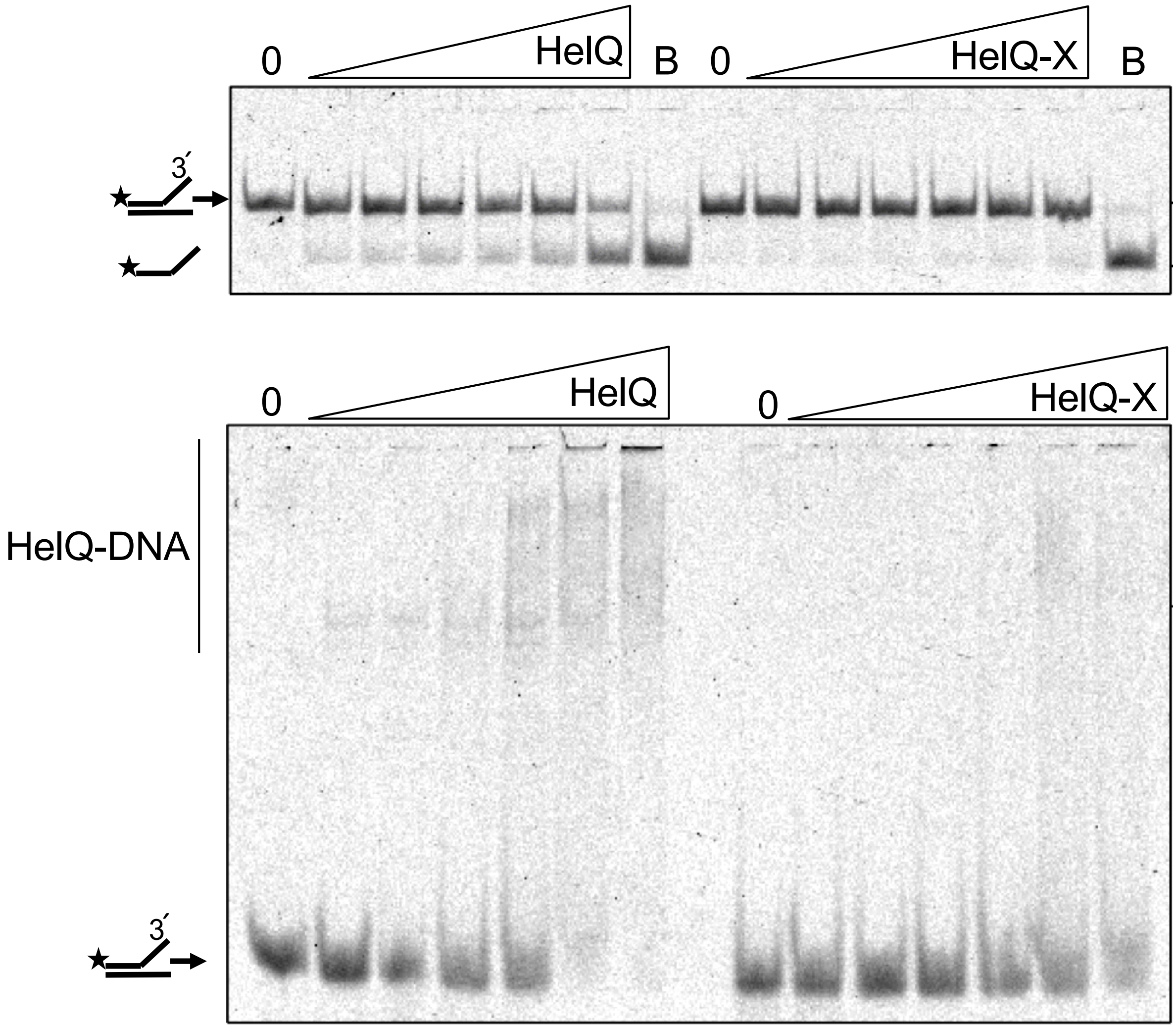

Figure S7

A.

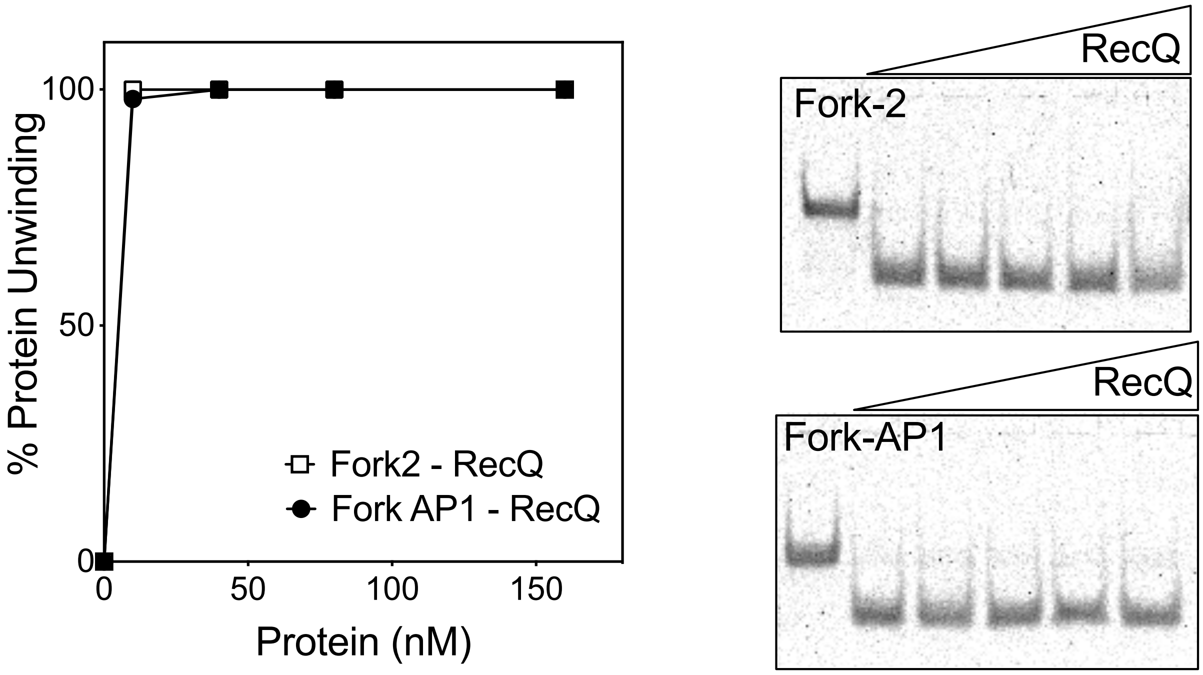

100
50
0
0
50
100
150
% Protein Unwinding
Protein (nM)
Fork2 - RecQ
Fork AP1 - RecQ
RecQ
Fork-2
RecQ
Fork-AP1

B.

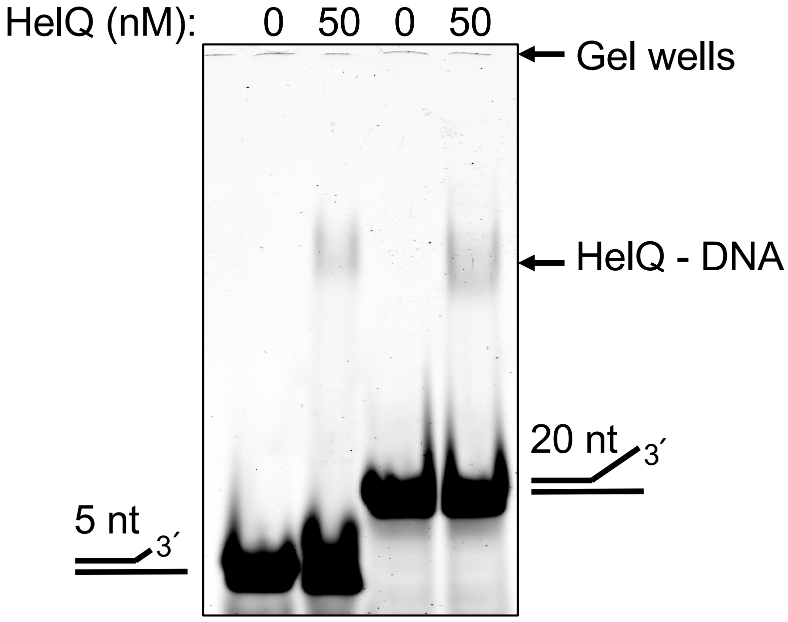

HelQ (nM):
0
50
0
50
Gel wells
HelQ - DNA
20 nt
3′
5 nt
3′

C.

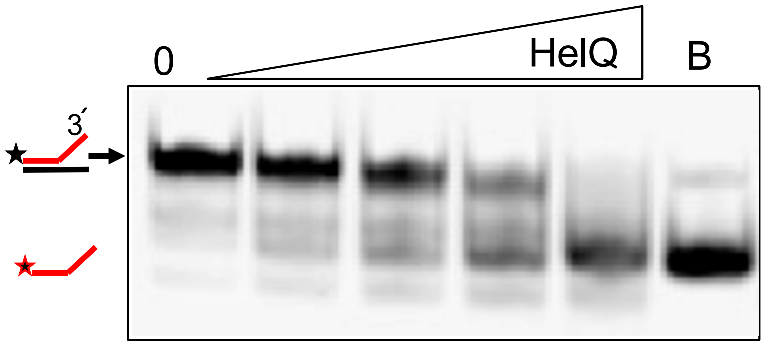

Figure S8

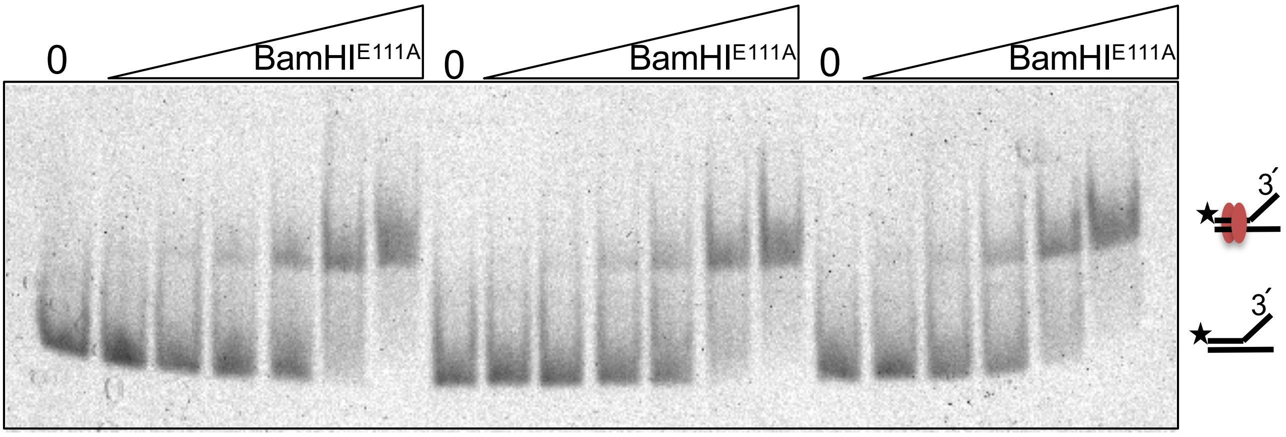

Figure S9

A. IUPred2A predictor

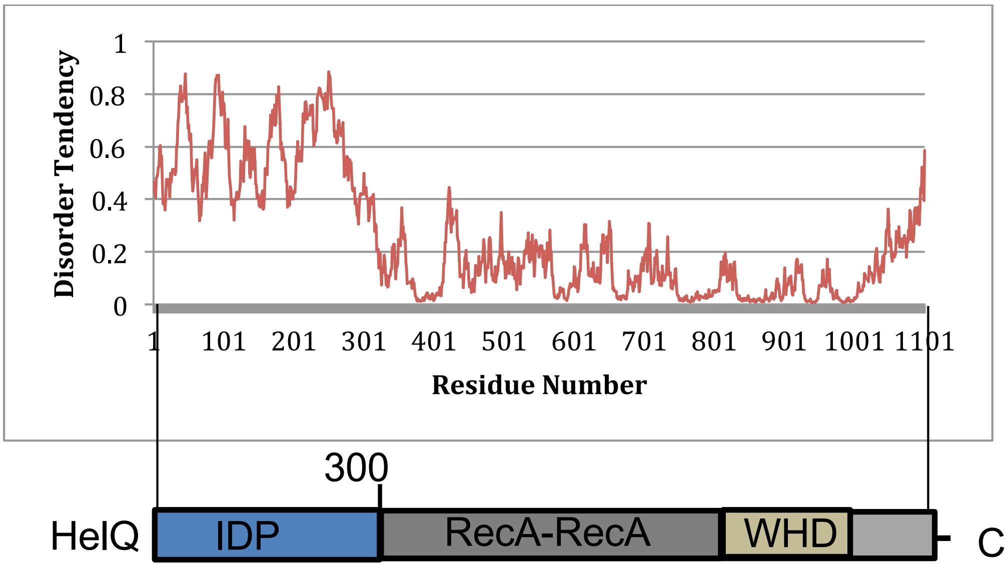

B. Sequence

The **highlighted portions of sequence** are where there is 75% agreement between all predictors in the database for this region being disordered.

>helq
MDECGSRIRRRVSLPKRNRPSLGCIFGAP**TAAELVPGDEGKEEEEMV**
AENRRRKTAGVLPVEVQPLLLSDSPECLVLGGGDTNPDLLRHMPTDR
GVGDQPN**DSEVDM**FGDYDSFTENSFIAQVDDLEQKYMQLPEHKKHAT
Conserved PWI Asp-Phe DFATENLCSESIKNKLSITTIGNLTELQ**TD**K**HTENQSGY**EGVTIEPG
ADLLYDVPSSQAIYFENL**QNSSNDLGDHSMKERDWKSSSHNTVNEEL**
End residue of N-HelQ **PHNCIEQPQQNDESSSKVRTSSDMNRRKSIKDHLKNA**MTGNAKAQTP
IFSRSKQLKDTLLSEEINVAKKTVESSSNDLGPFYSLPSKVRDLYAQ
FKGIEKLYEWQHTCLTLNSVQERKNLIYSLPTSGGKTLVAEILMLQE
LLCCRKDVLMILPYVAIVQEKISGLSSFGIELGFFVEEYAGSKGRFP
PTKRREKKSLYIATIEKGHSLVNSLIETGRIDSLGLVVVDELHMIGE
GSRGATLEMTLAKILYTSKTTQIIGMSATLNNVEDLQKFLQAEYYTS
QFRPVELKEYLKINDTIYEVDSKAENGMTFSRLLNYKYSDTLKKMDP
DHLVALVTEVIPNYSCLVFCPSKKNCENVAEMICKFLSKEYLKHKEK
EKCEVIKNLKNIGNGNLCPVLKRTIPFGVAYHHSGLTSDERKLLEEA
YSTGVLCLFTCTSTLAAGVNLPARRVILRAPYVAKEFLKRNQYKQMI
GRAGRAGIDTIGESILILQEKDKQQVLELITKPLENCYSHLVQEFTK
GIQTLFLSLIGLKIATNLDDIYHFMNGTFFGVQQKVLLKEKSLWEIT
VESLRYLTEKGLLQKDTIYKSEEEVQYNFHITKLGRASFKGTIDLAY
CDILYRDLKKGLEGLVLESLLHLIYLTTPYDLVSQCNPDWMIYFRQF
SQLSPAEQNVAAILGVSESFIGKKASGQAIGKKVDKNVVNRLYLSFV
LYTLLKETNIWTVSEKFNMPRGYIQNLLTGTASFSSCVLHFCEELEE
FWVYRALLVELTKKLTYCVKAELIPLMEVTGVLEGRAKQLYSAGYKS
LMHLANANPEVLVRTIDHLSRRQAKQIVSSAKMLLHEKAEALQEEVE
ELLRLPSDFP**GAVASST**DK**A**

Figure S10

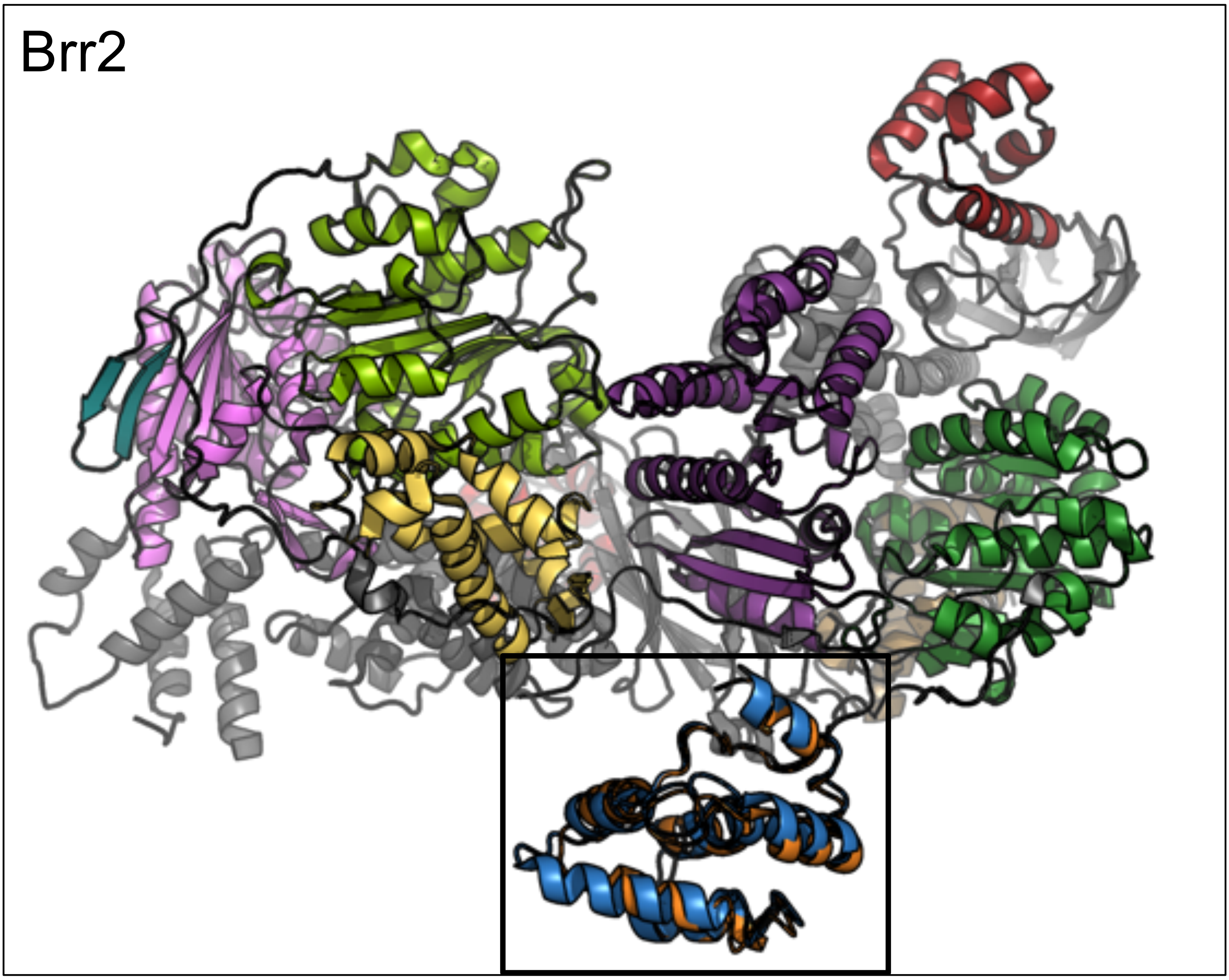

Figure S11

A.

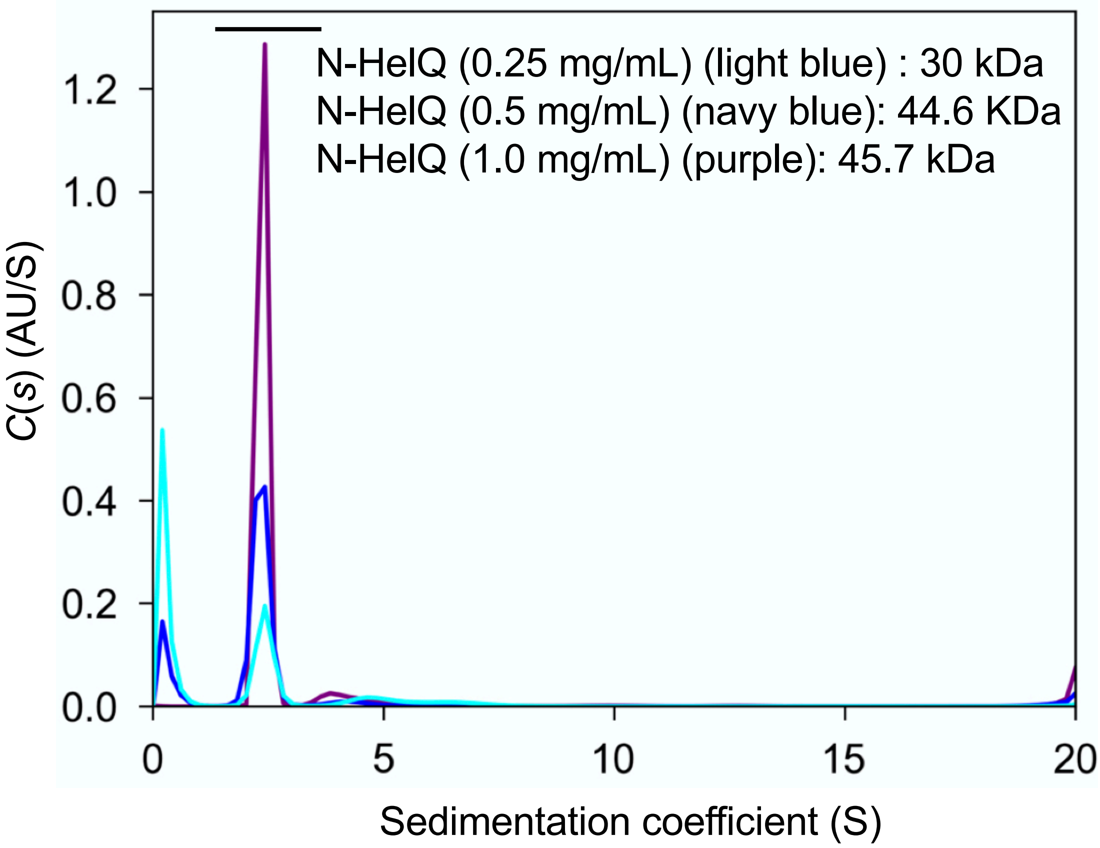

N-HelQ (0.25 mg/mL) (light blue) : 30 kDa
N-HelQ (0.5 mg/mL) (navy blue): 44.6 KDa
N-HelQ (1.0 mg/mL) (purple): 45.7 kDa
1.2
1.0
0.8
0.6
0.4
0.2
0.0
C(s) (AU/S)
0
5
10
15
20
Sedimentation coefficient (S)

Figure S11 (cont.)

B.

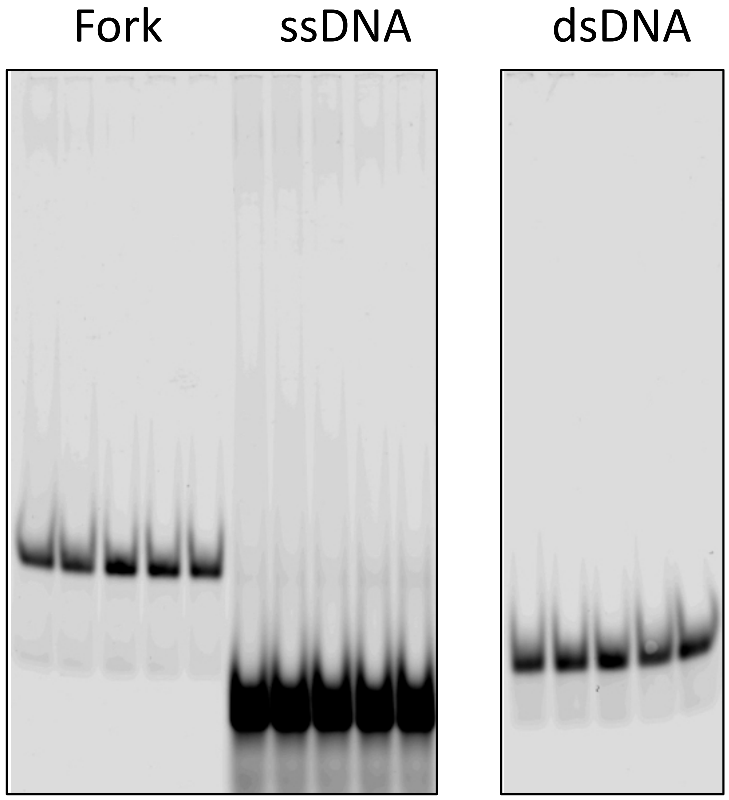

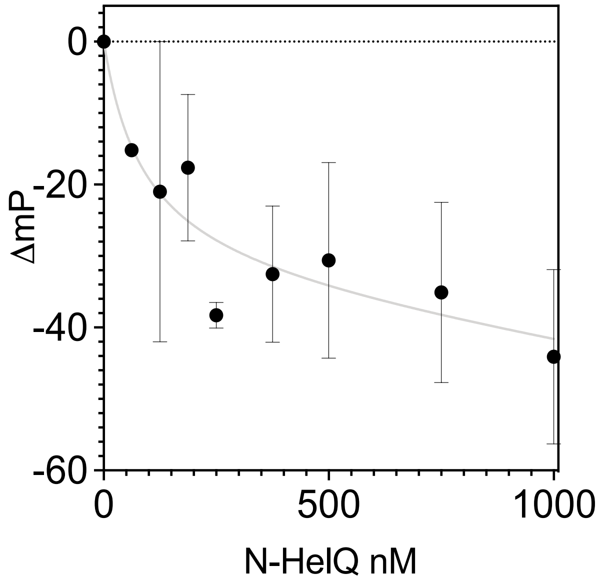

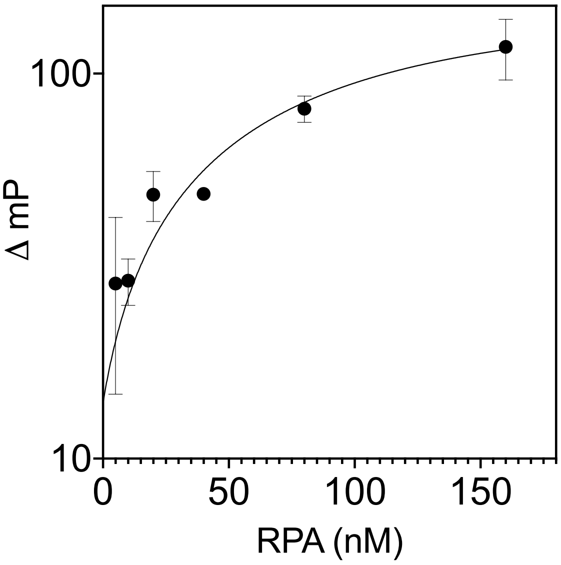

Figure S12

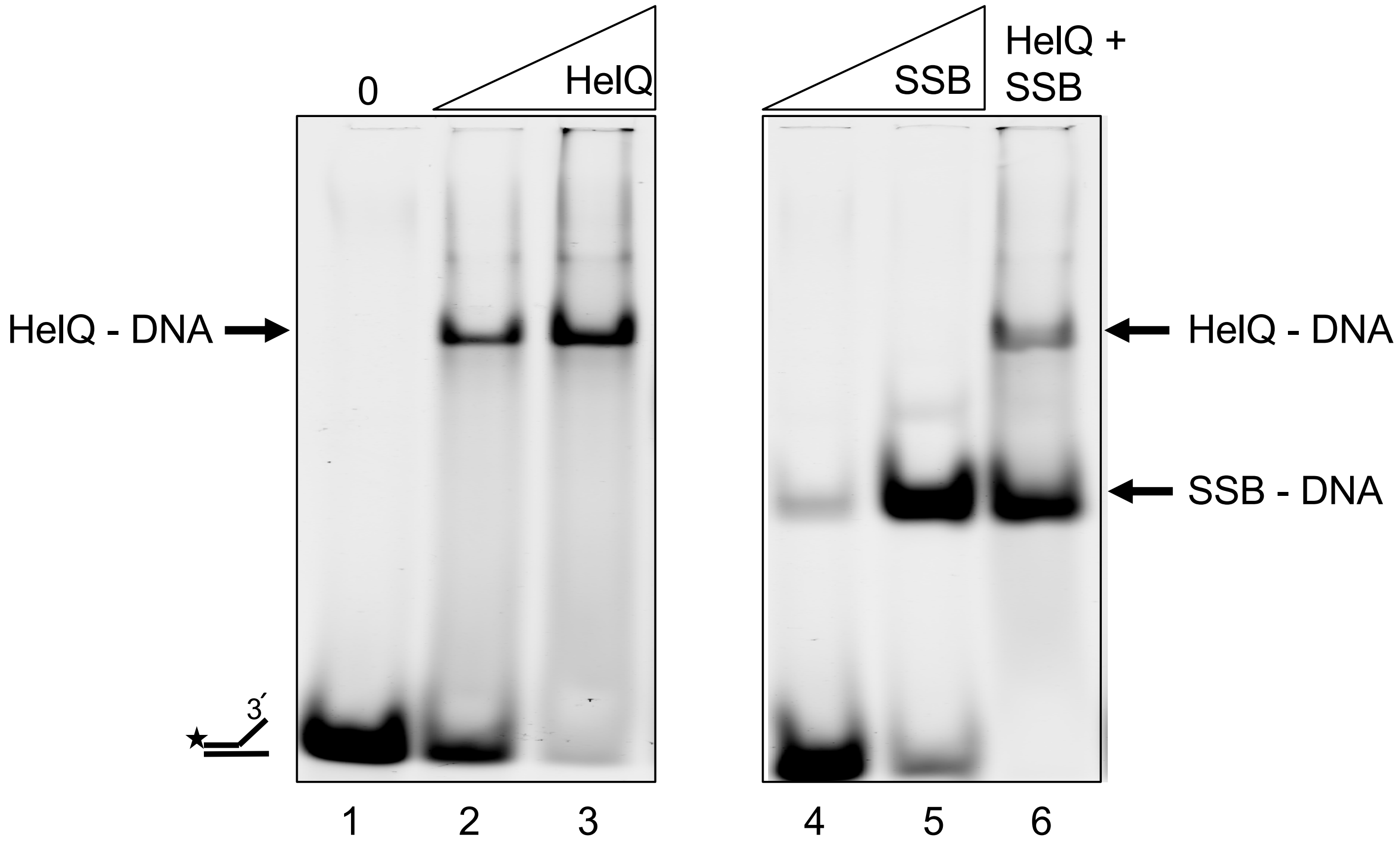

Figure S13

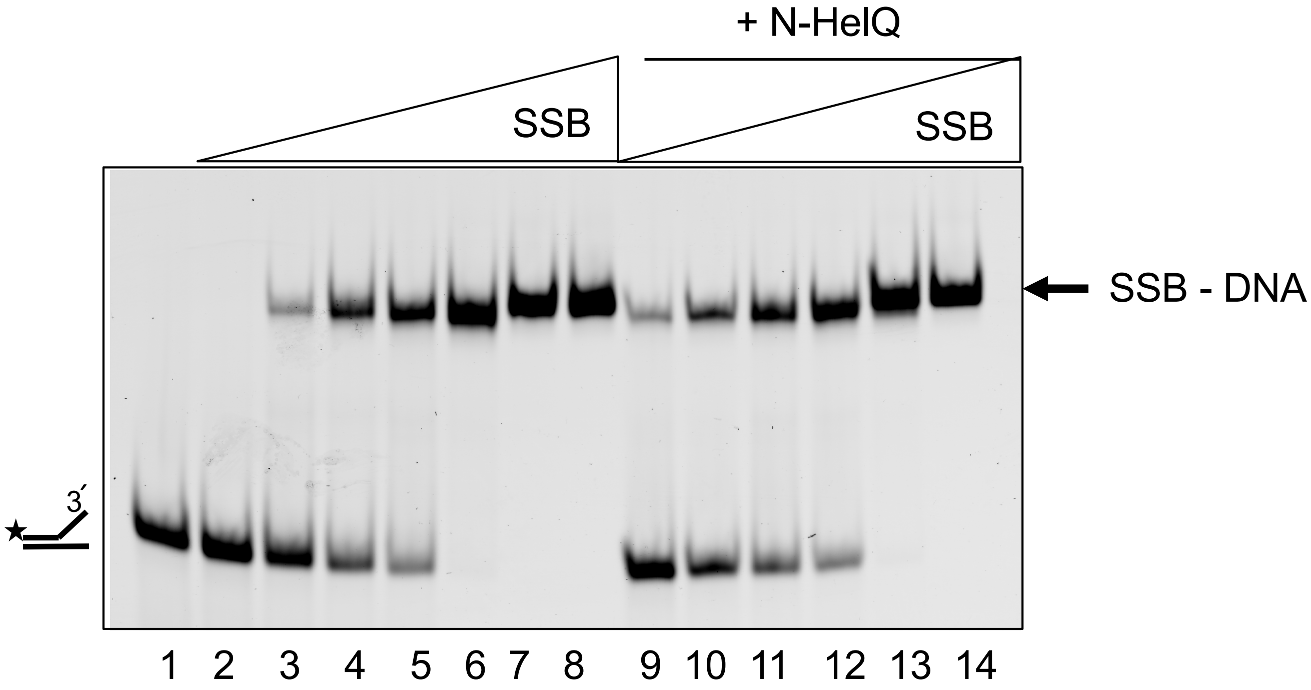

Figure S14

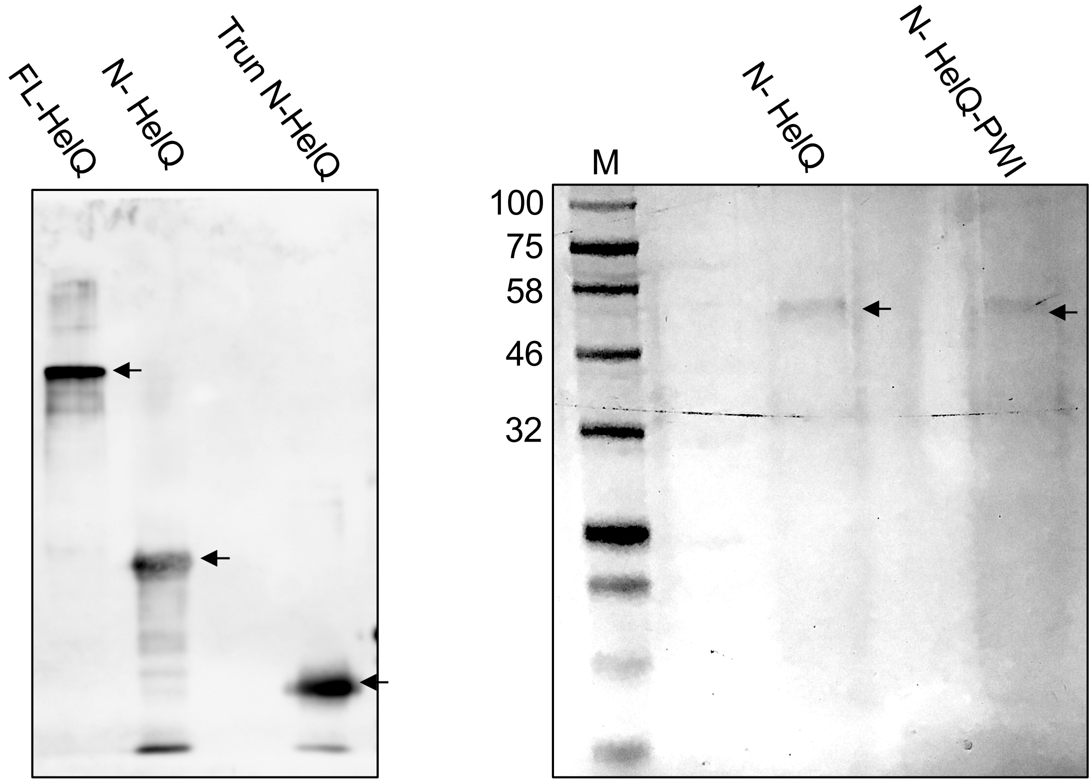

Figure S15

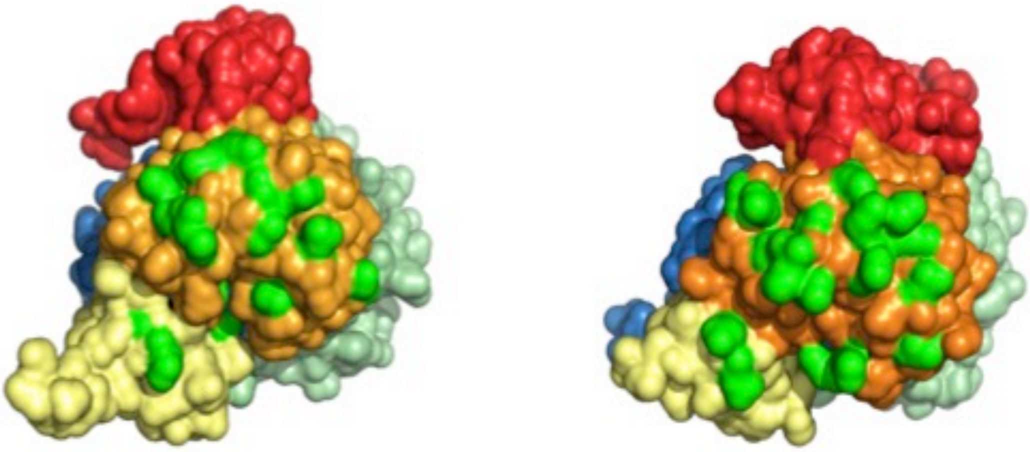

A: Phyre 2 HsaHelQ model

B: HsaPolQHD atomic resolution structure

Figure S16

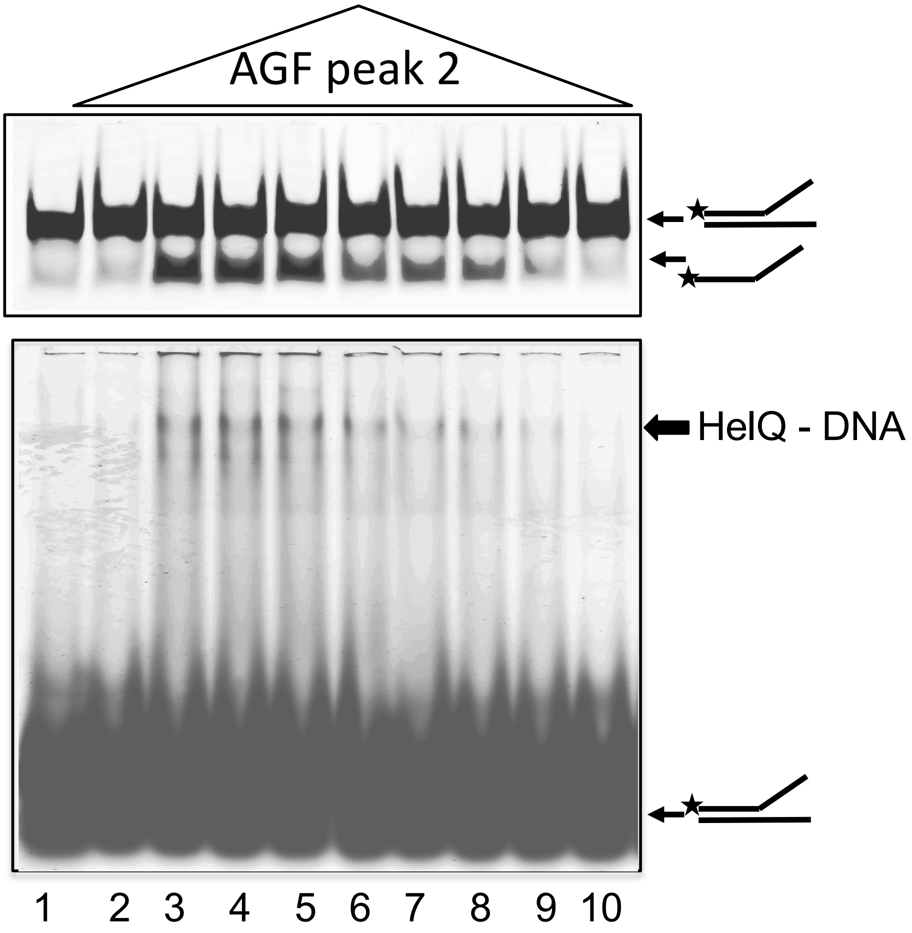

Figure S17

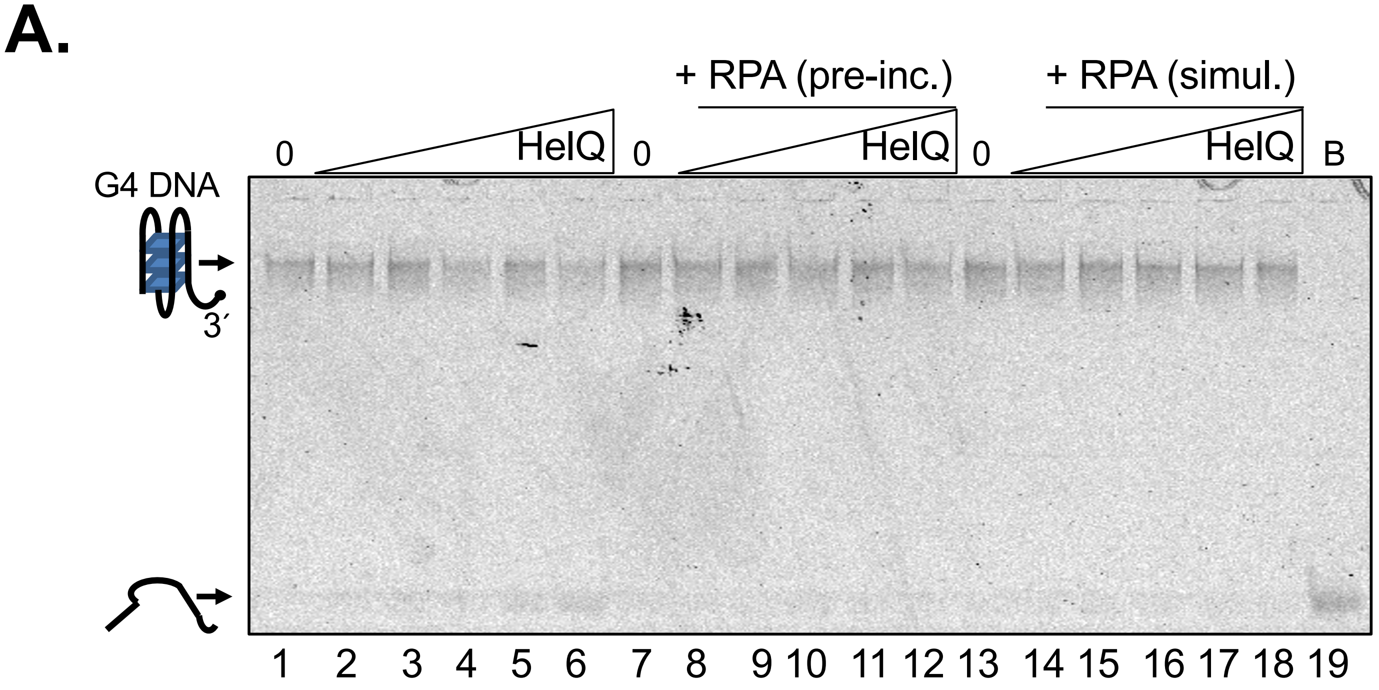

A.
+ RPA (pre-inc.)
+ RPA (simul.)
0
HelQ
0
HelQ
0
HelQ
B
G4 DNA
3´
1 2 3 4 5 6 7 8 9 10 11 12 13 14 15 16 17 18 19

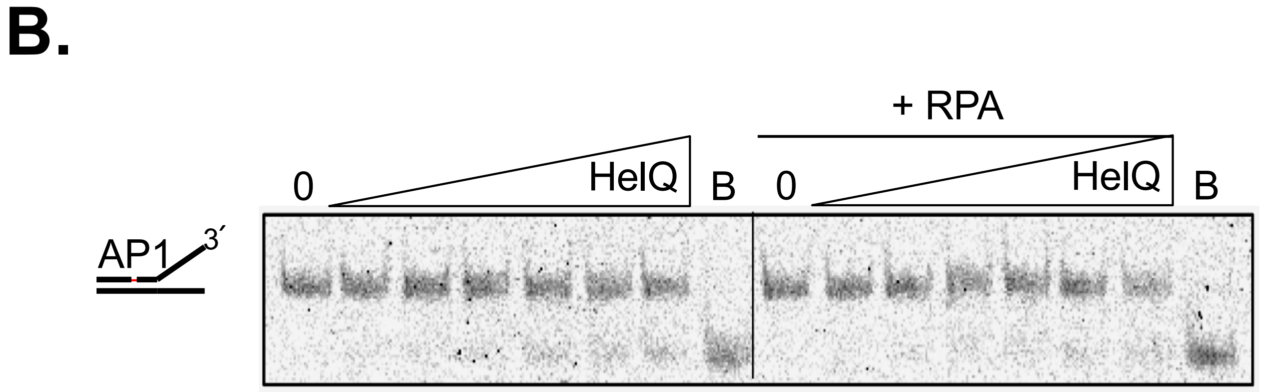

B.
+ RPA
0
HelQ
B
0
HelQ
B
AP1
3´
