# Supplementary material for: The HelQ human DNA repair helicase utilizes a PWI-like domain for DNA loading through interaction with RPA, triggering DNA unwinding by the HelQ helicase core
Source: NAR Cancer. 2021 Jan 12;3(1):zcaa043. doi: 10.1093/narcan/zcaa043 (PMC8210318; doi:10.1093/narcan/zcaa043)
Supplement: zcaa043_Supplemental_Files [file zcaa043_supplemental_files.zip › NARC - HelQ - Suppl. Text - R2.docx]

**Table S1. Peptides confirmed in C-HelQ**

| GATLEMTLAK | Q8TDG4 | 474 | 483 | HELQ | Helicase POLQ-like | Yes | Yes | 2 | 0.007155 |  | 1 | 4.53E+06 |
| --- | --- | --- | --- | --- | --- | --- | --- | --- | --- | --- | --- | --- |
| SLYIATIEK | Q8TDG4 | 432 | 440 | HELQ | Helicase POLQ-like | Yes | Yes | 2 | 0.028223 |  | 1 | 7.21E+06 |

**Table S2.** **AGF elution volumes for molecular mass standards.** These gave an equation for the line that was used to estimate HelQ protein masses:

Y = -4.786X + 37.32

| **Protein** | **Size** | **LOG (MW)** | **UV (mAU)** | **Elution Volume** | **Kav** |
| --- | --- | --- | --- | --- | --- |
| Ovalbumin | 43000 | 4.63346846 | 295.848 | 14.89 | 0.416025641 |
| Conalbumin | 75000 | 4.87506126 | 364.672 | 14.16 | 0.369230769 |
| Aldolase | 158000 | 5.19865709 | 322.529 | 12.62 | 0.270512821 |
| Ferritin | 440000 | 5.64345268 | 327.616 | 10.42 | 0.129487179 |
| Thyroglobulin | 669000 | 5.82542612 | 468.429 | 9.22 | 0.052564103 |

**Supplementary Figure Legends**

**Figure S1. Details of the forked DNA structures used in this work. A.** Chemically modified forks were as follows; forks-AP1, AP2 and AP3 were modified with a single abasic site where indicated (red), fork-Me by a single methylphosphonate as indicated, fork-S with a single phosphorothioate as indicted. Fork-A was used for binding to BamHI^E111A^ at the cognate sequence indicated. **B.** Additional details about each chemical modification in the context of a DNA duplex.

**Figure S2. Isolation of the N-terminal region of HelQ (N-HelQ).** The panel shows coomassie stained SDS-PAGE gel of degrading human HelQ (lane ‘HelQ’) when over-expressed and purified from Sf9 insect cells for 72 hours, highlighting a fragment that was isolated and identified by mass spectroscopy (shown below gel) as a 47 kDa N-HelQ fragment, comprising the first 240 amino acids of HelQ. This was used to clone, over-express and purify the N-HelQ fragment in *E. coli*.

**Figure S3. Heterotrimeric RPA purified from *E. coli.*** Coomassie stained SDS-PAGE of untagged RPA trimer comprises RPA70 (70 kDa), RPA32 (32 kDa) and RPA 14 (23.5 kDa). RPA was purified as described in the methods using sequential columns of Blue-Affi Gel, hydroxyapatite (HAP), MonoQ and superdex 200.

**Figure S4. HelQ D463A Walker B mutant is inactive as a helicase**. TBE gel showing helicase products from fork-2 DNA (25 nM) (see Figure S1) unwound by wild type HelQ (20, 40 and 80 nM) but not by HelQ^D463A^ at the same concentrations. Reactions in lanes labelled ‘0’ contained no protein and ‘B’ were boiled.

**Figure S5. Sequence homology of HelQ with the Ski2 helicase Hel308 used to generate ‘core’ helicase protein C-HelQ.** **A.** When metazoan HelQ amino acid sequences are excluded from pBlast searches, the human HelQ sequence shows highest homology to Hel308 from the euryarchaea. Homology is confined to a core HelQ region from, approximately, amino acid 350 – 800. **B.** This was confirmed by pairwise alignment in CLUSTAL although extending the region of homology to approximately amino acids 300 – 900.

**Figure S6. Chemical crosslinking inactivated HelQ.** The top panel shows products from unwinding of fork-2 by HelQ or HelQ-X (both 0, 5, 10, 20, 40, 80 and 160 nM) – the latter was HelQ treated with 0.2% glutaraldehyde crosslinking agent overnight at 4^o^C. Lane labelled ‘B’ was a boiled reaction to fully dissociate fork-2 into ssDNA, and the star indicates the position of the 5’ Cy5-DNA end label. The bottom panel is an EMSA of the same reactions.

**Figure S7. (A). *E. coli* RecQ helicase used as a positive control for unwinding DNA through a single abasic site that inhibited human HelQ helicase.** Shown are a graph for RecQ unwinding either fork-2 or fork-AP1 (each at 25 nM), and representative gels used for measurements in duplicate with standard error bars shown. **(B).** HelQ (50 nM) binding to fork-2.05 (5 nt overhang) and fork 2.20 (20 nt overhang) are very similar despite being unable to unwind fork-2.05. The sample wells of the gel are indicated to note that HelQ is forming distinct protein-fork DNA complexes not general protein-DNA aggregates. **(C). HelQ translocates RNA to unwind an RNA-DNA hybrid.** Summary of HelQ **(**0, 10, 20, 40, 80 and 160 nM) unwinding a forked substrate (25 nM) comprised of a translocating RNA strand in an RNA-DNA hybrid as indicated. Lane labelled ‘B’ was a boiled reaction to fully dissociate the fork into ssDNA and ssRNA, and the position of the Cy5 end label is indicated with a star.

**Figure S8. BamHI^E111A^ bound to DNA fork-A shown in EMSAs in triplicate.** Reactions contained 25 nM of the fork DNA, and BamHI^E111A^ at 10, 20, 40, 0, 80 and 160 nM at 24^o^C for 10 minutes. This established that 100% of the forked DNA was bound by BamHI^E111A^ at 160 nM, for utilisation in helicase assays. A star indicates the position of the 5’ Cy5-end label.

**Figure S9.** **N-HelQ** **is predicted to contain several regions of intrinsic protein disorder.** **A.** Summarises the predicted tendency to protein disorder made by IUPred2A database across the full 1101 amino acids of HelQ, identifying high tendency corresponding to 300 amino acid N-terminal region (N-HelQ) labelled as an intrinsically disordered protein (IDP). **B.** Highlights two major areas of protein predicted disorder within N-HelQ (bold font) and their positions relative to a predicted PWI structural fold and conserved motif detailed in the main results.

**Figure S10. Crystal structure of the *S. cerevisiae* Brr2 protein** (PDB code 5DCA) highlighting (inset box) the PWI-like domain identified in the N-terminal region of this protein, superimposed with a model for HelQ as in the main results Figures 4A and 4I.

**Figure S11**. **N-HelQ is monomeric in analytical ultracentrifugation (AUC). A.** AUC was carried out on a Beckman ProteomeLab XL AUC system. AUC was measured at an absorbance of 0.2-1 OD_600_ to test a protein_._ N-HelQ was dialysed into 20 mM Tris pH 8. Three concentrations of N-HelQ were tested: 0.25, 0.5 and 1 mg/mL at room temperature as indicated in the graph. **B. N-HelQ does not bind to DNA. B.** N-HelQ (50, 100, 200 and 500 nM) did not show any evidence for stable DNA complex formation in EMSAs mixed with fork-2, duplex DNA or ssDNA substrates, each at 25 nM. Duplex DNA was formed from fork-2 strand-1 annealed to its complement, and ssDNA was fork-2 strand 1. Fork-2 DNA binding of N-HelQ was also assessed in comparison with RPA using fluorescence anisotropy. N-HelQ (0-1000 nM) and RPA (0-160 nM) were incubated with 4 nM fluorescein-labelled fork DNA to measure change in polarisation at 0, 5 and 10 minutes after addition of protein. Reactions were repeated three times and plotted showing standard error from the mean values.

**Figure S12. HelQ does not form ‘super-shift’ complex in EMSAs when added to pre-bound SSB-DNA complex.** HelQ (0, 80, 160 nM, lanes 1-3) or SSB alone (45 or 90 nM, lanes 4 and 5) bound to fork-2 (25 nM) as a single major complex with different electrophoretic mobilities, the same complexes as observed when HelQ (160 nM) was added to SSB-DNA (90 nM) complex (lane 6).

**Figure S13. N-HelQ does not displace *E. coli* SSB-DNA complexes.** In EMSAs SSB (0-300 nM) added to fork-2 DNA (25 nM) (lanes 1-8), was not dissociated by addition of N-HelQ at 500 nM (lanes 9-14).

**Figure S14. N-HelQ and mutants were purified stably from *E. coli.*** Summary of N-HelQ and mutants used in this work. Anti-His 1^o^ immuno-blot analysis of N-HelQ (47.5 kDa) and truncated N-HelQ (28.2 kDa) sizes in comparison to FL-HelQ (141 kDa). Coomassie stained SDS PAGE gel of WT-N-HelQ and PWI N-HelQ with mutations Asp-142 and Phe-143 at 47.5 kDa.

**Figure S15. Structural modelling and amino acid sequence analysis of HelQ compared to its closest human homologue, the helicase region of Polθ, predicts oligomerisation interfaces of HelQ to cluster in helicase ‘ratchet’ domain 4.** Human HelQ (HsaHelQ) and PolQ (HsaPolQHD), a structural model and the published atomic resolution structure (PDB 5AGA) respectively, are indicated by domain: domain 1 (RecA like), blue; domain 2 (RecA like), green; domain 3 (winged helix domain), yellow; domain 4 (helicase ratchet), orange; domain 5, light green. The interaction interfaces were obtained for PolQ from PDB 5AGA. EMBOSS Water local alignments were made between PolQ and HelQ, giving residues conserved between the two proteins that participate in PolQ monomer-monomer interactions, highlighted in green. These are predicted to cluster on predicted domain 4 of HelQ, the helicase ‘ratchet’ highlighted in orange. The HelQ model was generated using Phyre2 from FASTA amino acid sequence obtained from UniProt accession number Q8TDG4. For clarity, amino acids 1-300 are omitted from HelQ because these cannot be modelled by Phyre2 because of lack of overall sequence similarity to other proteins.

**Figure S16. ATP-activated HelQ binds to DNA in EMSAs.** The top panel is a duplicate of Figure 1H to which is added an EMSA in which 10 ul of the same AGF fractions used for the top panel, in buffer +ATP-Mg^2+^, were mixed with Cy5-end labelled fork DNA (25 nM), to detect HelQ-DNA complexes corresponding to peak fork unwinding.

**Figure S17. RPA heterotrimer does not stimulate HelQ to unwind through G4 or abasic DNA barriers. A.** HelQ (0, 10, 20, 40, 80 and 160 nM) was unable to dissociate G4 DNA (lanes, 1-6, see also main Figure 3), and this is not improved by addition of RPA (25 nM) when either pre-incubated with G4 DNA (lanes 7-12) or added simultaneously to DNA with HelQ (lanes 13-17). Lane 18 shows fully dissociated G4 DNA from a boiled reaction sample. **B.** Inhibition of HelQ (0, 5, 10, 20, 40, 80 and 160 nM) fork DNA unwinding by a single abasic site in fork-AP1 (see also main Figure 3) was not alleviated by addition of RPA (25 nM) that was pre-incubated with the fork DNA, as indicated. Dissociation of the fork is shown by boiling a reaction sample indicated by ‘B’.
